# Loss of histone H3.3 results in DNA replication defects and altered origin dynamics in *C. elegans*

**DOI:** 10.1101/gr.260794.120

**Published:** 2020-12

**Authors:** Maude Strobino, Joanna M. Wenda, Laura Padayachy, Florian A. Steiner

**Affiliations:** Department of Molecular Biology and Institute for Genetics and Genomics in Geneva, Section of Biology, Faculty of Sciences, University of Geneva, 1211 Geneva, Switzerland

## Abstract

Histone H3.3 is a replication-independent variant of histone H3 with important roles in development, differentiation, and fertility. Here, we show that loss of H3.3 results in replication defects in *Caenorhabditis elegans* embryos at elevated temperatures. To characterize these defects, we adapt methods to determine replication timing, map replication origins, and examine replication fork progression. Our analysis of the spatiotemporal regulation of DNA replication shows that despite the very rapid embryonic cell cycle, the genome is replicated from early and late firing origins and is partitioned into domains of early and late replication. We find that under temperature stress conditions, additional replication origins become activated. Moreover, loss of H3.3 results in altered replication fork progression around origins, which is particularly evident at stress-activated origins. These replication defects are accompanied by replication checkpoint activation, a delayed cell cycle, and increased lethality in checkpoint-compromised embryos. Our comprehensive analysis of DNA replication in *C. elegans* reveals the genomic location of replication origins and the dynamics of their firing, and uncovers a role of H3.3 in the regulation of replication origins under stress conditions.

To ensure rapid and uniform duplication of the genome during the cell cycle, DNA replication is initiated in a bidirectional way at many different sites in the genome, called replication origins. Replication initiation is composed of two nonoverlapping steps called “origin licensing” and “origin firing.” Origin licensing occurs during G1 through binding of the origin recognition complex (ORC) and the recruitment of the replication licensing factors Cdt1 and Cdc6, which together load the minichromosome maintenance protein complex (Mcm2-7) (Sonneville et al. 2012). Origin firing occurs upon entry into S phase, through binding of different initiation factors like Sld2 (RecQL4/RecQ4 in humans), Sld3 (TICRR in humans) and Dpb11 (TopBP1 in humans), and phosphorylation of the Mcm2-7 complex (Labib and Diffley 2001; Kanemaki and Labib 2006; Bruck and Kaplan 2015). These firing factors are present in limiting amounts (Mantiero et al. 2011; Tanaka et al. 2011), and origins therefore fire at different times during S phase, resulting in large domains of early and late replication (MacAlpine et al. 2004; Farkash-Amar et al. 2008; Desprat et al. 2009; Dileep and Gilbert 2018; Takahashi et al. 2019).

Under replication stress conditions (e.g., conflicts between transcription and replication forks, depletion of nucleotides, or depletion of histones), replication forks can stall or collapse (Groth et al. 2007; Bester et al. 2011; Técher et al. 2017; Macheret and Halazonetis 2018). Replication stress can activate the ATR checkpoint, which phosphorylates the checkpoint kinase 1 (Chk1), leading to cell cycle delay or arrest (Liu et al. 2000; Hyun et al. 2016; Moiseeva et al. 2019). As a response to moderate replication stress, cells can activate additional origins (Técher et al. 2017), called latent or dormant origins, that are inactive during the undisturbed S phase (Woodward et al. 2006; Boos and Ferreira 2019). How firing origins are selected over dormant origins and under what conditions dormant origins are activated is unclear (Fragkos et al. 2015).

Numerous assays have been developed to map replication origins in different organisms (Prioleau and MacAlpine 2016), mostly by mapping different replication elements such as nascent DNA strands (Besnard et al. 2012), replication bubbles (Mesner et al. 2013), Okazaki fragments (Petryk et al. 2016), replication initiation sites (Langley et al. 2016), or initiation factors (Dellino et al. 2013; Miotto et al. 2016). Despite these efforts, the number, location, and dynamic regulation of replication origins remains controversial. For example, two studies in *Caenorhabditis elegans* recently provided genome-wide maps of replication origins using two different methods, Okazaki fragment sequencing and nascent DNA strand sequencing, and arrived at largely different origin numbers and conclusions (Pourkarimi et al. 2016; Rodríguez-Martínez et al. 2017). Additional approaches are therefore required to understand how origins are defined and dynamically regulated.

The variant histone H3.3 has been linked to different steps of DNA replication. It is enriched at replication origins in *Drosophila melanogaster* and *Arabidopsis thaliana* (Deal et al. 2010; MacAlpine et al. 2010; Eaton et al. 2011; Stroud et al. 2012; Paranjape and Calvi 2016), and loss of H3.3 results in a slower replication fork in chicken cells (Frey et al. 2014). H3.3 is associated with early replication domains and is recruited to sites of DNA repair in human cells (Adam et al. 2013; Clément et al. 2018). Loss of H3.3 results in lethality or sterility in most organisms (Hödl and Basler 2009; Sakai et al. 2009; Jang et al. 2015; Tang et al. 2015; Wollmann et al. 2017). The *C. elegans* genome contains five genes encoding H3.3 homologs, and we recently showed that knockout of all five genes resulted in viable worms with a reduced number of viable offspring at higher temperatures (Delaney et al. 2018). In *C. elegans*, elevated temperature can lead to increased mutation rate, reduced fecundity, and increased sensitivity to chemicals (Matsuba et al. 2013; Cedergreen et al. 2016). Temperature affects cell division timing and developmental robustness, and wild-type worms show cell division defects and sterility at temperatures >25°C (Richards et al. 2013; Begasse et al. 2015; Neves et al. 2015). Given the role of H3.3 in DNA replication in other organisms, we speculated that the reduced brood size observed in *C. elegans* H3.3-null mutants at elevated temperatures is caused by defects in DNA replication. We therefore aimed to investigate the role of H3.3 in DNA replication and origin firing and to adapt tools to study these processes to the *C. elegans* model system.

## Results

### Genome-wide replication timing in *C. elegans* embryos

**Figure 1.**
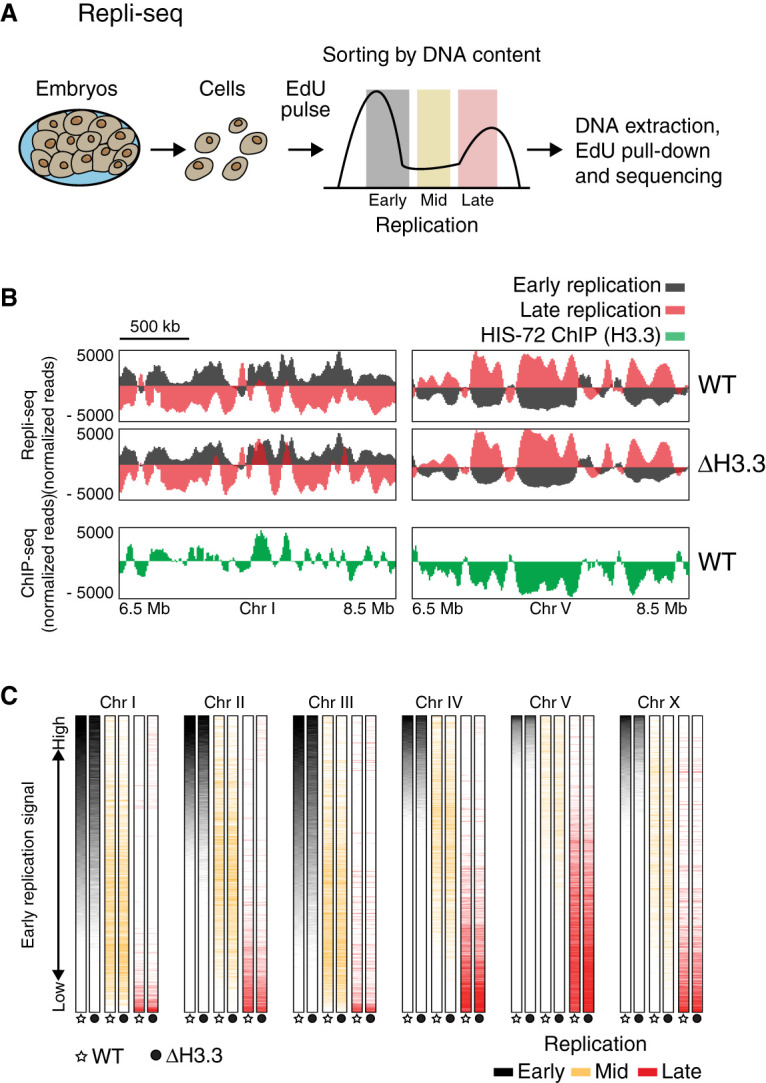
Replication timing is unchanged in the absence of H3.3. (*A*) Schematic description of Repli-seq. Embryonic cells were dissociated, exposed to an EdU pulse for 5 min, and sorted according to their DNA content. EdU-labeled DNA was sequenced and mapped to the genome. (*B*) Representative genome browser views of Repli-seq and H3.3 ChIP-seq. Repli-seq signal is shown for wild-type (WT) and H3.3-null mutant (Δ H3.3) worms on regions of Chromosomes I and V. Early S phase is shown in black and late S phase in red. HIS-72 (H3.3) ChIP-seq signal is shown in green for the same regions. ChIP-seq data from Delaney et al. (2019). (*C*) Color-coded replication timing for each chromosome. Repli-seq signal from early (black), mid (orange), and late (red) S phase for WT and Δ H3.3. Data for each chromosome were sorted according to the signal of early S phase in WT.

We first determined whether loss of H3.3 causes global changes in the temporal program of genome replication. To characterize replication timing during the rapid cell cycle in early *C. elegans* embryogenesis, we adapted Repli-seq for use in this organism (Hansen et al. 2010). This method relies on the incorporation of the thymidine analog 5-ethynyl-2′-deoxyuridine (EdU) into newly replicated regions of the genome (Fig. 1A). To separate early and late replicated genomic regions, we pulse-labeled asynchronous embryonic cell populations with EdU, then sorted the cells according to their DNA content and sequenced EdU-containing fragments. We obtained cells that just entered S phase (early replication), showed ongoing DNA synthesis (mid replication), or were at or near completion of replicating the genome (late replication) (Supplemental Fig. S1A). These Repli-seq experiments revealed that the genome is partitioned into mutually exclusive domains of early and late replication (Fig. 1B,C), and that different chromosomes display distinct overall replication timing patterns. Domains of early replication are enriched on Chromosomes I, II, and III, whereas domains of late replication are more prevalent on Chromosomes IV, V, and X (Fig. 1C; Supplemental Fig. S1B). Following the hypothesis that H3.3 has a role in DNA replication, we compared the replication domains to H3.3 incorporation and found that domains of early replication correlate well with the presence of HIS-72, the most highly expressed *C. elegans* H3.3 homolog (Fig. 1B; Supplemental Fig. S1C). However, H3.3-null mutant worms showed largely the same early and late replication domains as wild-type worms, indicating that H3.3 is not a major driver of global DNA replication timing (Fig. 1B,C; Supplemental Fig. S1B,D). Genes in domains of early replication show higher levels of expression compared to genes in domains of later replication, consistent with the observation from other organisms that regions of open chromatin replicate early (Supplemental Fig. S1E; Prioleau and MacAlpine 2016; Ekundayo and Bleichert 2019).

### Mapping replication origins by ChEC-seq and EdU-seq

**Figure 2.**
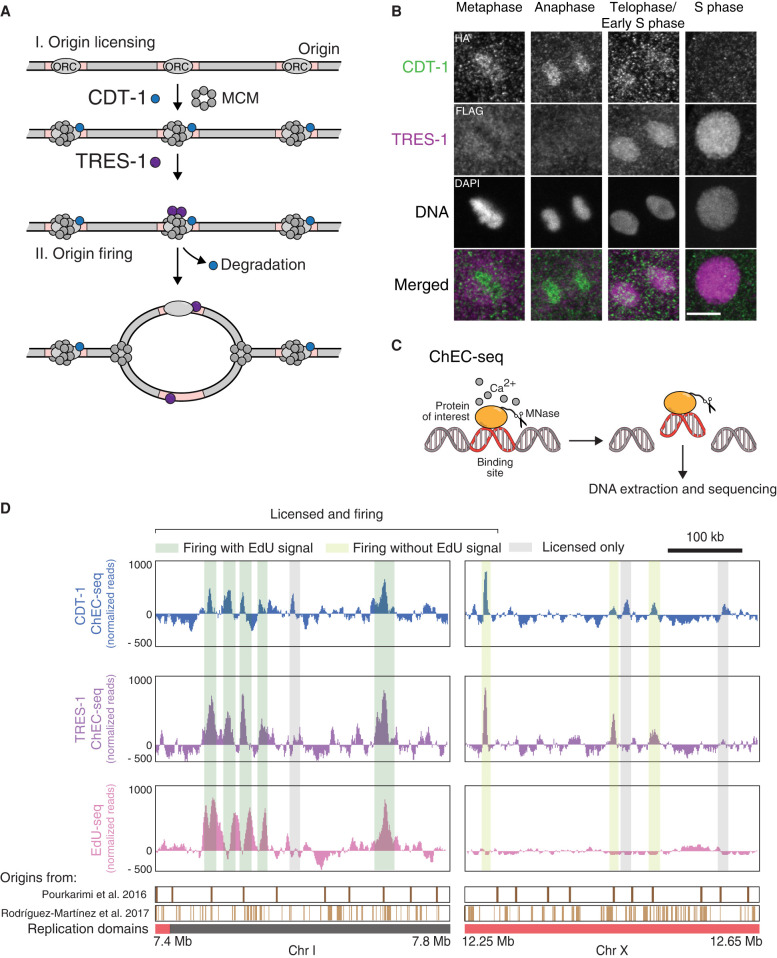
Identification of replication origins in *C. elegans* embryos. (*A*) Schematic description of the roles of CDT-1 and TRES-1 in replication origin firing. CDT-1 is required for the licensing of all origins. TRES-1 is recruited only to origins that fire. (*B*) Localization of HA::CDT-1 (green) and FLAG::TRES-1 (magenta) during the cell cycle. Immunofluorescence images using anti-HA and anti-FLAG antibodies and DAPI are shown. Scale bar represents 5 µm. (*C*) Schematic description of ChEC-seq. The protein of interest is fused with MNase. Upon activation with calcium in purified nuclei, MNase cleaves and releases DNA fragments at the binding sites of the fusion proteins. These small fragments are isolated and sequenced. (*D*) Representative genome browser views of CDT-1 ChEC-seq (blue), TRES-1 ChEC-seq (purple), and EdU-seq (pink) signal. Identified origins are highlighted in gray (licensed only) or dark and light green (licensed and firing with and without EdU signal, respectively). Domains of late (red) and early (gray) replication and positions of replication origins identified in previous studies are shown *below* the genome browser (Pourkarimi et al. 2016; Rodríguez-Martínez et al. 2017).

We next aimed to define replication origins and their timing during S phase. Given the enrichment of H3.3 at replication origins in other organisms, we speculated that loss of H3.3 may alter the position or timing of origin firing. Previous origin mapping approaches in *C. elegans* do not allow for the analysis of origin dynamics during the cell cycle (Pourkarimi et al. 2016; Rodríguez-Martínez et al. 2017). We therefore took an alternative approach that combines the mapping of replication origin factors and the observation of nucleotide incorporation at origins. We first profiled the genomic binding sites of two conserved proteins, CDT-1 (CDT1 in humans) and TRES-1 (TICRR in humans), that are involved in replication origin licensing and firing, respectively (Fig. 2A; Zhong et al. 2003; Kumagai et al. 2010; Sonneville et al. 2012; Gaggioli et al. 2020). Although CDT-1 is only a prerequisite for origin licensing (binding of the MCM complex), and the function of TRES-1 in *C. elegans* has not been characterized until very recently, we consider binding of CDT-1 and TRES-1 as evidence for licensing and firing, respectively. Consistent with these roles, CDT-1 is present on chromatin during anaphase and telophase, but levels become undetectable during S phase, and TRES-1 appears on chromatin during telophase but remains present during S phase (Fig. 2B). Typically, genomic protein binding sites are profiled by chromatin immunoprecipitation. However, this approach depends on antibody availability and solubility of the chromatin component of interest, both of which can be limiting. To circumvent these problems, we adapted chromatin endogenous cleavage (ChEC) to *C. elegans*. This method relies on the fusion of the protein of interest with micrococcal nuclease (MNase), which cuts DNA around the binding sites of the fusion protein and releases DNA fragments that can be sequenced and mapped (Fig. 2C; Schmid et al. 2004; Zentner et al. 2015). We generated MNase fusion constructs at the endogenous *cdt-1* and *tres-1* loci using CRISPR-Cas9, resulting in worms that are phenotypically wild type. To generate genome-wide binding maps of CDT-1 and TRES-1, we performed ChEC-seq experiment on purified embryonic nuclei and identified peaks of CDT-1 and TRES-1 signal. We observed peaks that are distributed across the genome in both data sets. Moreover, as expected, the TRES-1 peaks mostly overlap with CDT-1 peaks (licensed and firing), but some CDT-1 peaks show no corresponding TRES-1 signal (licensed only) (Fig. 2D). The peaks are wider than the expected footprint of the proteins, but it is unclear if this reflects local variability of the protein binding or the range within which DNA is accessible to the MNase of the fusion proteins.

To identify replication origins that fire immediately after entry into S phase, we modified the Repli-seq protocol. Instead of analyzing asynchronous cell populations, we used hydroxyurea (HU) to synchronize cells. Upon HU treatment, most cells are arrested at the entry of the S phase, and a large part of the cell population proceeds with DNA replication upon removal of HU (Supplemental Fig. S2). We combined the release into S phase with a second HU block in the presence of EdU, allowing the cells to replicate short stretches of DNA around early replication origins before arresting. The EdU-containing DNA fragments were then isolated and sequenced. These EdU-seq experiments revealed discrete peaks that are located in close proximity to a subset of the CDT-1 and TRES-1 peaks (licensed and firing with EdU signal) (Fig. 2D). The EdU-seq signal sometimes appears as double peaks flanking the CDT-1 and TRES-1 peaks, indicating that we detect short fork movements away from the replication origins.

### Classification of origins genome-wide

**Figure 3.**
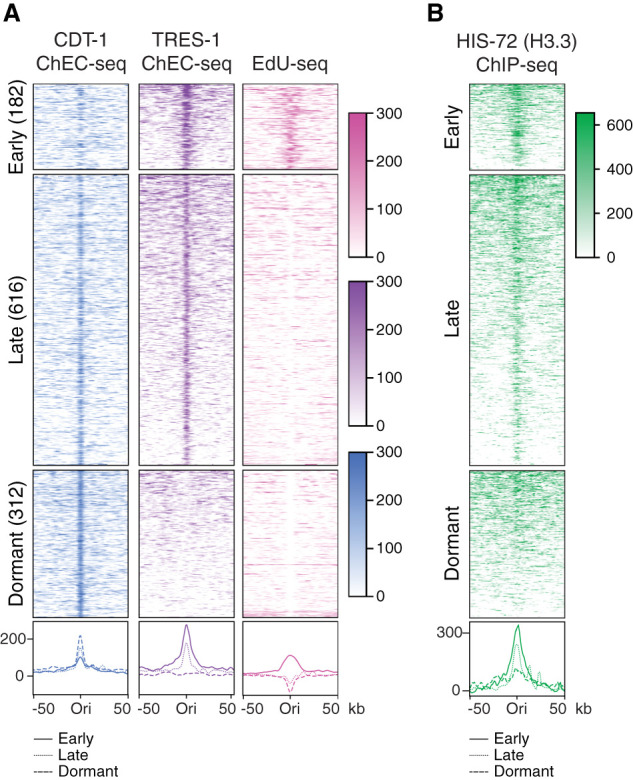
Classification of replication origins in *C. elegans* embryos. (*A*) Heatmaps (*top*) and average plots (*bottom*) of CDT-1 ChEC-seq (blue), TRES-1 ChEC-seq (purple), and EdU-seq (pink) signal at replication origins. Origins were identified by peak calling using the CDT-1 ChEC-seq, TRES-1 ChEC-seq, and EdU-seq data sets obtained from worms grown at 20°C and classified into early, late, and dormant origins through unsupervised clustering. Signal (normalized reads) is shown for a 50-kb window around each origin. (*B*) Heatmaps (*top*) and average plots (*bottom*) of HIS-72 (H3.3) ChIP-seq signal (Delaney et al. 2019) at replication origins, as in *A*.

To obtain a map of embryonic replication origins genome-wide, we combined the peak calls from the CDT-1 ChEC-seq, TRES-1 ChEC-seq, and EdU-seq experiments and obtained a map of 1110 origins. Based on the signal in the three data sets, we used unsupervised clustering to classify “early firing,” “late firing,” and “dormant” origins (Fig. 3A). Early firing origins are defined by enrichment in all three data sets, as they are licensed (CDT-1) and fire at the onset of S phase (TRES-1 and EdU-seq). Late firing origins are defined by enrichment in CDT-1 and TRES-1 ChEC-seq data sets, but not EdU-seq, because they fire after the onset of S phase. Dormant origins are defined by enrichment only in the CDT-1 ChEC-seq data set, because they are licensed but do not fire under standard conditions (Fig. 3A; Supplemental Fig. S3A). We did not obtain any clusters with TRES-1 signal in absence of CDT-1 signal. Although the enrichment of CDT-1 and TRES-1 overlaps within a narrow window at the replication origins, the distribution of EdU-seq signal is more broad, which likely reflects short stretches of fork progression away from the replication origins after the release from the initial HU block (Supplemental Fig. S3B). Firing origins are found mostly outside of gene-coding regions, whereas dormant origins are enriched within genes (Supplemental Fig. S3C). Similar to *D. melanogaster* and *A. thaliana* (Deal et al. 2010; MacAlpine et al. 2010; Eaton et al. 2011; Stroud et al. 2012; Paranjape and Calvi 2016), H3.3 is enriched around the origins of all three categories (Fig. 3B; Supplemental Fig. S3D), suggesting that it may play a role in origin identity or activation.

### Altered origin dynamics in H3.3-null mutants

**Figure 4.**
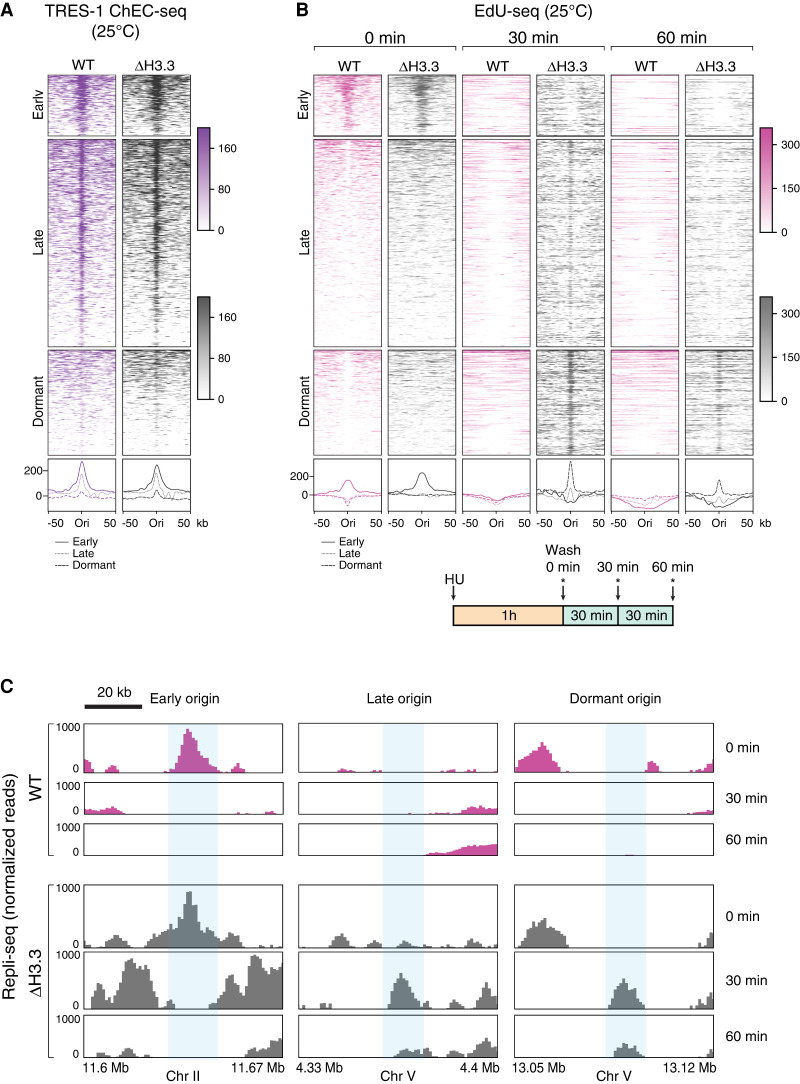
Replication origin dynamics are altered in H3.3-null mutants. (*A*) Heatmaps (*top*) and average plots (*bottom*) of TRES-1 ChEC-seq signal at replication origins, for wild-type (WT, purple) and H3.3-null mutant (Δ H3.3, gray) worms grown at 25°C. Signal is shown for a 50-kb window around each origin. (*B*,*C*) EdU-seq time course, for WT (pink) and Δ H3.3 (gray) worms grown at 25°C. (*B*) Heatmaps (*top*) and average plots (*bottom*) of EdU-seq signal at 0, 30, and 60 min at all origins. Signal (normalized reads) is shown for a 50-kb window around each origin. The cartoon shows the experimental protocol, in which cells were incubated 1 h with HU, released from the HU block, and treated with EdU and HU at 0, 30, or 60 min (indicated by the asterisks). (*C*) Genome browser views of EdU-seq signal at 0, 30, and 60 min, showing fork progression at representative examples of early, late, and dormant origins.

We next compared the distribution of firing origins in WT and H3.3-null mutant worms. We repeated the TRES-1 ChEC-seq and the EdU-seq experiments at 25°C, in which a difference in brood size between the two strains is observed. The TRES-1 ChEC-seq experiments showed that the origins licensed in WT worms were also used in absence of H3.3 (Fig. 4A; Supplemental Fig. S4A). Additionally, in both strains, we observed weak TRES-1 signal at origins that are dormant at 20°C (Fig. 4A; Supplemental Fig. S4). These origins were identified solely based on the presence of CDT-1 under normal conditions, and the presence of TRES-1 at 25°C shows that these are functional origins that become activated at higher temperatures. This result confirms the presence of “backup” origins that are inactive during undisturbed S phase, but are activated under replication stress (Woodward et al. 2006; Boos and Ferreira 2019). We did not detect any difference between the firing of early origins in WT and H3.3-null mutant based on EdU-seq signal (Fig. 4B, 0 min). These results indicate that the origin distribution and the firing of early origins remains unchanged upon loss of H3.3.

We next examined if fork progression following origin firing is affected by the loss of H3.3. The EdU-seq method not only allows one to identify early firing origins, but can also be used to visualize fork progression around these origins by adding an EdU pulse at 0, 30, or 60 min after the release from the HU block (Fig. 4B,C; Supplemental Fig. S5A–C). As expected, the EdU signal enrichment at the early firing origins is no longer present after 30 min, and regions occupied by early firing origins incorporate very little EdU after 60 min, which reflects the bidirectional movement of the replication forks away from the origins (Fig. 4B,C; Supplemental Fig. S5A–C). This bidirectional fork movement is difficult to observe for late origins, because we have no means to synchronize their firing. Nevertheless, the EdU-seq signal at late origins appears depleted at 30 min, suggesting that they fire within the first 30 min after S-phase onset. In H3.3-null mutants, as described above, early origins appear to fire normally, and we observe bidirectional fork movement. However, we found that the EdU signal persists around origins after 30 and 60 min, consistent with the fork progression being abnormal (Fig. 4B,C; Supplemental Fig. S5A–C). This altered fork progression appears at origins of all classes and results in an enrichment of EdU incorporation at late origins. The difference between WT and H3.3-null mutant cells is even more pronounced at origins that are dormant under standard conditions. These origins fire in both strains at 25°C, as evidenced by the increased presence of TRES-1 compared to 20°C (Figs. 3A, 4A; Supplemental Fig S4B), but fork progression from the origins is impaired in H3.3-null mutants, resulting in an accumulation of EdU-seq signal at later time points. The enrichment at late and dormant origins is observed within a narrow window (peak width at half height is 3 kb for late and 5 kb for dormant origins, compared to 17 kb for early origins at *t* = 0 min) (Supplemental Fig. S5D), suggesting that the forks progress differently and may stall or collapse immediately after origins fire. Alternatively, the abnormal fork progression in H3.3-null mutants may result from defects in fork restart following HU-induced fork arrest. The coincidence of this EdU-seq signal enrichment with the center of late and dormant origins is particularly noteworthy, because these sites were identified solely based on a completely independent method—namely, the mapping of the CDT-1 and TRES-1 binding sites. The aberrant fork progression in H3.3-null mutant worms is only seen under mild temperature stress conditions and is not observed at 20°C (Supplemental Fig. S6), indicating that H3.3 is required for fork progression or origin dynamics under temperature stress.

### Fork progression or restart, but not fork speed, is affected by loss of H3.3

**Figure 5.**
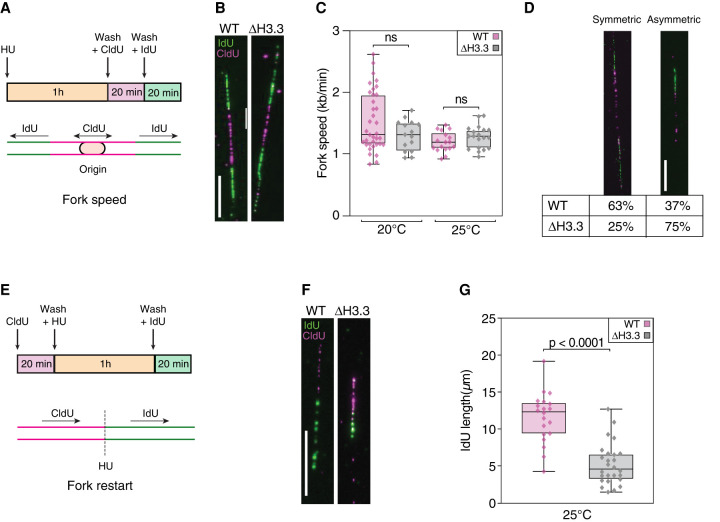
Replication fork progression or restart, but not speed, is affected by loss of H3.3. (*A*) Cartoon of the experimental protocol used to measure fork speed and the frequency of fork stalling. Cells were synchronized by HU during 1 h before washes and incubation with CldU for 20 min and IdU for 20 min, allowing the observation of bidirectional fork movement at origins. (*B*) Representative examples of DNA combing images used to measure replication fork speed for wild-type (WT) and H3.3-null mutants (Δ H3.3). (*C*) Fork speed determined by DNA combing at 20°C and 25°C for WT and Δ H3.3. (*D*) Representative examples and percentages of symmetric (*left*) and asymmetric (*right*) forks in WT and Δ H3.3 at 25°C detected by DNA combing. (*E*) Cartoon of the experimental protocol used to assess fork restart after HU treatment. Cells were incubated with CldU for 20 min, HU for 1 h, and IdU for 20 min, allowing detection of fork restart after fork arrest by HU treatment. (*F*) Representative examples of DNA combing images used to assess fork restart after HU treatment for WT and Δ H3.3 at 25°C. (*G*) Quantification of IdU incorporation after HU treatment for WT and Δ H3.3 at 25°C. IdU incorporation is shown in green and CldU incorporation in magenta; scale bars represent 10 µm or 20 kb in all DNA combing panels. Significance was tested using unpaired *t*-tests.

To establish if loss of H3.3 resulted in a slowing of the replication fork speed along the chromatin fiber, we adapted DNA combing to *C. elegans* (Michalet et al. 1997). This method relies on the pulse labeling of DNA by the subsequent incorporation of two thymidine analogs, CldU and IdU. By visualizing the length of IdU incorporation on stretched individual chromatin fibers, replication fork speed can be estimated (Fig. 5A). We performed DNA combing experiments using embryonic cells synchronized at the entry of S phase. We first assessed the fork speed at replication origins, identified by a stretch of CldU incorporation with adjacent bidirectional IdU incorporation (Fig. 5B). We found that fork speed is ∼1.5 kb/min, but did not detect any significant difference between WT and H3.3-null mutant embryos (Fig. 5C). We next looked for evidence of stalled forks around the origins, which are evident by asymmetric IdU signal around the central stretch of CldU incorporation. We found that such asymmetric IdU incorporation appears at higher frequency in H3.3-null mutant worms compared to WT (Fig. 5D). To test whether this asymmetry resulted from increased fork stalling or defects in fork restart, we modified the DNA combing protocol to artificially induce fork arrest using HU in unsynchronized cells, followed by detection of IdU incorporation (Fig. 5E). We found that H3.3-null mutant cells showed significantly shorter stretches of IdU incorporation compared to WT cells, indicating that they are defective in fork restart after fork stalling (Fig. 5F,G).

### Delayed cell cycle and checkpoint activation in H3.3-null mutant embryos

**Figure 6.**
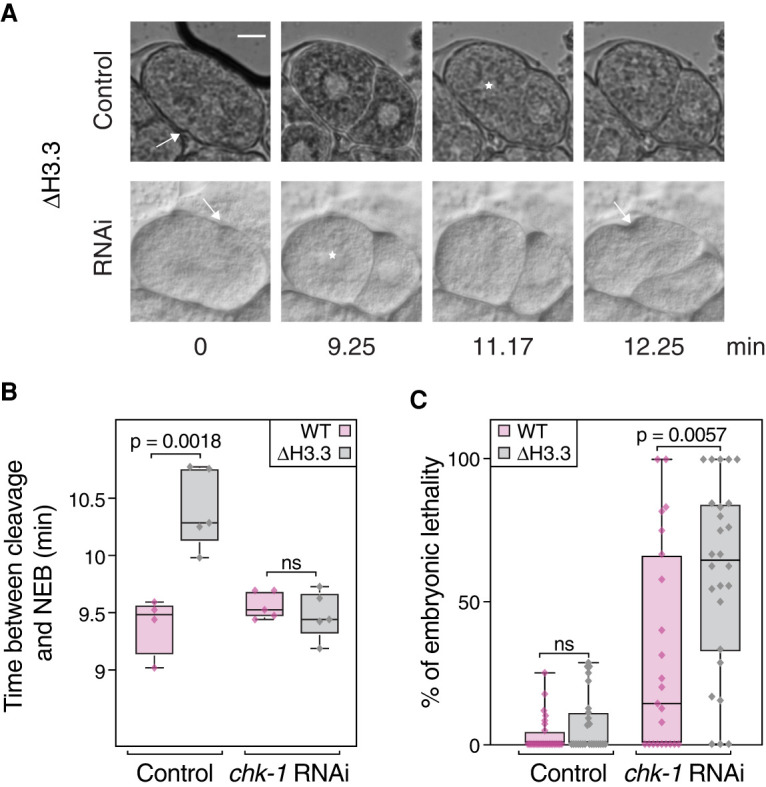
Loss of H3.3 results in cell cycle delays and replication checkpoint activation. (*A*) Determination of cell cycle length from the onset of the cleavage furrow of the first cell division (arrow) to nuclear envelope breakdown of the AB cell (star). Representative still images from a movie of a developing H3.3-null mutant (Δ H3.3) embryo with and without depletion of *chk-1* by RNAi are shown. Scale bar represents 10 µm. (*B*) Cell cycle timing for WT and Δ H3.3 worms, with and without depletion of *chk-1* by RNAi. (*C*) Embryonic lethality for WT and Δ H3.3, with and without depletion of *chk-1* by RNAi. Significance was tested using unpaired *t*-tests.

In *C. elegans* embryos, DNA replication defects are characterized by an increased length of the first embryonic cell cycle (Brauchle et al. 2003; Holway et al. 2006; Stevens et al. 2016). We measured cell cycle timing from the appearance of a cleavage furrow in the P0 cell to nuclear envelope breakdown of the AB cell at 25°C (Fig. 6A). We found that it was significantly increased in H3.3-null mutant embryos compared to WT (Fig. 6B). Removing the checkpoint kinase 1 (CHK-1) by RNAi restored normal cell cycle timing, suggesting that the cell cycle delay is linked to a replication defect (Fig. 6A,B). Moreover, removal of CHK-1 resulted in an increase of the embryonic lethality in H3.3-null mutants (Fig. 6C). Together, our results indicate that loss of H3.3 results in defects in DNA replication or DNA damage repair, and that these defects become detrimental upon removal of CHK-1.

## Discussion

The variant histone H3.3 has previously been linked to DNA replication, but its mechanistic role in this process and how its function relates to its genomic localization are not well understood. Our recent discovery that *C. elegans* H3.3-null mutants are viable allowed us to analyze potential developmental defects in more detail (Delaney et al. 2018). Here, we describe that under temperature stress conditions, H3.3 is required for DNA replication dynamics around replication origins and protects the genome from replication stress.

For understanding the replication defects upon loss of H3.3, we first had to develop and adapt tools to investigate DNA replication dynamics in *C. elegans* embryos. Our adaptation of the Repli-seq method revealed that despite the very short cell cycle during embryogenesis, genomic regions of early and late replication are mutually exclusive and distributed in a nonrandom fashion (Fig. 1; Supplemental Fig. S1). Partitioning of the genome into early and late replicating domains is well described for mammalian genomes, both in cell population averages (MacAlpine et al. 2004; Farkash-Amar et al. 2008; Hiratani et al. 2008; Desprat et al. 2009) and single cells (Dileep and Gilbert 2018; Takahashi et al. 2019). The domains of early replication are enriched for H3.3 in *C. elegans*, as it has been observed in human cells (Clément et al. 2018). We found that loss of H3.3 does not globally alter the domains of early and late replication (Fig. 1; Supplemental Fig. S1). Local differences between WT and H3.3-null mutant worms are sometimes observed (Fig. 1B), but the genome-wide correlation suggests that they are rare (Supplemental Fig. S1D). This is maybe not surprising, because replication timing has been found to be very robust and remains unaltered in a wide range of mutants that affect chromatin organization (Marchal et al. 2019).

To comprehensively identify replication origins throughout S phase, we combined methods that detect the genomic binding sites of proteins involved in origin identity (the origin licensing factor CDT-1 and the origin firing factor TRES-1) with methods that rely on the identification of sites of nucleotide incorporation during origin firing (EdU-seq) (Figs. 2, 3). This approach allows the identification of origin firing dynamics during the cell cycle and also includes dormant origins that act as backup origins for use under specific conditions. *C. elegans* origins have been mapped previously using Okazaki fragment sequencing or nascent strand sequencing, both of which identify firing origins (Pourkarimi et al. 2016; Rodríguez-Martínez et al. 2017). Our approach identified significantly fewer origins (∼1000 vs. ∼2000 or ∼16,000, respectively), but the genomic locations of the origins identified in this study largely overlap with those previously identified (Supplemental Fig. S7A). We analyzed CDT-1, TRES-1, and EdU-seq levels at the origins identified in previous studies and found that a large proportion of the origins identified by Okazaki fragment sequencing showed enrichment for CDT-1 and TRES-1, but this was less pronounced for the majority of origins identified by nascent strand sequencing (Supplemental Fig. S7B). We independently estimated the number of origins required to replicate the entire genome in our system based on the speed of the replication fork and by determining the time required to replicate the entire genome {Origins = genome size [kb]/(2× time required for genome replication [min] × fork speed [kb/min])}. We found that EdU incorporation of cells synchronized by HU plateaus ∼40 min after the first observed EdU incorporation (Supplemental Fig. S7C). This is substantially longer than the S-phase duration observed in developing embryos (Sonneville et al. 2012), and we attribute the delay to the dissociation of cells during isolation and the treatment with HU and EdU. Based on this replication time, the genome size and the fork speed measured in the DNA combing experiment, we estimate the number of firing origins required for replicating the entire genome to be 859. This number is very close to the number of firing origins identified by ChEC-seq and EdU-seq (798). Although our methods allow for the detection of origins that only fire under specific conditions, they still rely on averaging the signal from large populations of cells and will therefore be less sensitive to detect origins that are only licensed at specific developmental time points or in specific cell types.

**Figure 7.**
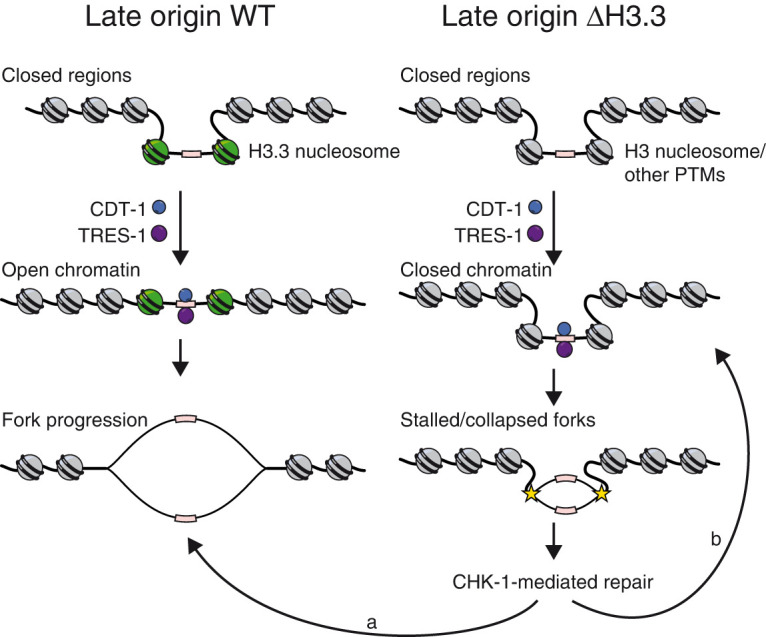
Model for the role of H3.3 at late firing origins. Late origins tend to be located in regions of closed chromatin. In WT (*left*), the presence of H3.3 may facilitate the opening of the origin upon activation and replication fork progression away from the origin or may allow H3.3 to be efficiently recruited to sites of fork stalling. Upon loss of H3.3 (Δ H3.3; *right*), the opening of the chromatin context or fork restart after fork stalling may be impaired, leading to delays in fork progression. The replication stress is sensed by the CHK-1 checkpoint and enables damage repair and fork restart (b) and eventual fork progression as in WT (a).

H3.3 is enriched at replication origins in *D. melanogaster* and *A. thaliana*, but the functional significance of this enrichment remains unclear (Deal et al. 2010; MacAlpine et al. 2010; Eaton et al. 2011; Stroud et al. 2012; Paranjape and Calvi 2016). We found that H3.3 is enriched at replication origins in *C. elegans*, but consistent with the findings from *D. melanogaster*, we found no evidence that loss of H3.3 inhibits replication origin firing (Figs. 3, 4; Paranjape and Calvi 2016). However, we found clear differences in fork progression around origins as S phase progresses, in particular, at those origins that only fire under stress conditions. The replication defects are evident both in meta-analysis (EdU-seq) (Fig. 4) and single molecule analysis (DNA combing) (Fig. 5), and can be indicative of an increased frequency of fork stalling or of defects in fork restart after induced fork stalling. We speculate that H3.3 is required for an efficient fork restart and may be recruited to stalled or collapsed forks, similar to what has been observed in culture cells for sites of DNA damage (Adam et al. 2013; Frey et al. 2014). H3.3 enrichment at replication origins may therefore not be linked to origin activity directly, but may serve as a pre-recruitment to these sites to be able to quickly resolve fork stalling and ensure appropriate origin firing (Fig. 7). The replication problems in H3.3-null mutant worms are counteracted by a CHK-1-dependent prolongation of the cell cycle and become detrimental when the checkpoint is removed (Fig. 6). This cell cycle delay is not visible in the Repli-seq experiments, as they rely on the cell sorting based on DNA content, which corrects for cell cycle timing differences. The replication defects are only observed at 25°C, and origin dynamics in wild-type and H3.3-null mutant worms are indistinguishable at 20°C, consistent with the fertility defects of H3.3-null mutant worms that are only observed at higher temperatures. The reasons behind the temperature sensitivity upon loss of H3.3 are currently unclear, but it may be linked to the faster cell cycle progression at 25°C compared to 20°C (Begasse et al. 2015; Neves et al. 2015). Consistent with this hypothesis, we found that the proportion of wild-type cells that actively incorporate EdU increases in embryos kept at 25°C compared to 20°C, whereas this proportion stays about the same for H3.3-null mutant cells at both temperatures (Supplemental Fig. S7D). The chromatin context of the replication origins may also influence their firing. The presence of H3.3 may facilitate the opening of origins upon activation to allow fork progression (Fig. 7). In *D. melanogaster* embryos, a decrease in histone concentration results in an increased cell cycle length and CHK-1 activation, similar to our observations in *C. elegans* embryos (Chari et al. 2019). We carried out MNase-seq experiments and found no evidence that the nucleosome positioning is altered upon loss of H3.3, suggesting that H3.3 is replaced by canonical H3 (Supplemental Fig. S7E). *C. elegans* replication origins were found to be enriched for H3 acetylation, H3K4 methylation, and H3K27 acetylation (Pourkarimi et al. 2016), and it is possible that the presence of H3.3 aids the deposition of some of these marks. However, the causal relationship between histone modifications and the identity of replication origins is not clear. DNA replication could also be indirectly affected by the loss of H3.3 through an altered transcriptional landscape genome-wide. However, we previously reported that loss of H3.3 only results in minor changes in the transcriptome (Delaney et al. 2018). More research will be needed to establish a causal relationship between the chromatin context and replication origin formation to understand how the timing of origin firing is regulated and to determine how the replication fork interacts with nucleosomes containing different histone modifications or variants.

## Methods

### Worm culture and strain generation and phenotype analysis

Worms were grown according to standard procedures at 20°C or 25°C. A list of the strains generated and used in this study is given in Supplemental Table S1. RNAi experiments were carried out as previously described (Kamath et al. 2003). For determining embryonic lethality at 25°C, worms were exposed to RNAi or control food overnight, and the number of eggs, hatched larvae, and adults was counted on subsequent days. For determining the cell cycle length during early embryonic cell divisions at 25°C, worms were exposed to RNAi or control food overnight. Adults were cut to release young embryos, which was recorded on a Leica DMI8 microscope, with the temperature maintained at 25°C. Cell cycle timing was measured from the appearance of the cleavage furrow of the P0 cell to nuclear envelope breakdown of the AB cell. For determining the subcellular localization of CDT-1 and TRES-1, embryos cut from adult worms were freeze-cracked, fixed in cold methanol (5 min), and stained using an anti-FLAG antibody (Sigma-Aldrich F7425.2MG) to detect FLAG::MNase::TRES-1 and using an anti-HA antibody (mAb 42F13, FMI Monoclonal Antibody Facility) to detect HA::MNase::CDT-1.

### Chromatin endogenous cleavage (ChEC) and MNase-seq

Embryos from synchronized adult populations were isolated with sodium hypochlorite and chitinase treated. Nuclei were released with a glass dounce homogenizer, separated from debris by centrifugation at 100*g*, and pelleted by centrifugation at 1000*g*. MNase was activated by the addition of CaCl_2_ to a final concentration of 2 mM. Samples were incubated for 5–10 min (for CDT-1 and TRES-1 ChEC) or for 1 h (free MNase control) at 30°C. For the control sample, 0.3 unit/mL of MNase (NEB) was added at the same time as the CaCl_2_. MNase was inactivated by adding an equal volume of 2× stop buffer (400 mM NaCl, 20 mM EGTA). Digested DNA was extracted for constructing the libraries using NEBNext Ultra II DNA Library Prep. Paired-end read sequencing reactions were performed on an Illumina HiSeq 2500 sequencer at the Genomics Platform of the University of Geneva.

A similar protocol was used for MNase-seq, with the following modifications. Samples were incubated with 0.1 unit/mL MNase for different times (30 sec to 10 min) to account for differential MNase-sensitivity of chromatin. DNA from the different digestion times was pooled before library construction. The MNase-seq profiles around origins were determined after in silico size selection of the fragments between 120 and 200 bp.

### Embryonic cell extraction for EdU incorporation or DNA combing

Cells were extracted from embryos as previously described (Bianchi and Driscoll 2006). Briefly, embryos were isolated using sodium hypochlorite and treated with chitinase. Cells were dissociated with a syringe and resuspended in supplemented L-15 medium.

### EdU-seq

EdU-seq was carried out as described (Macheret and Halazonetis 2019) with minor modifications. Embryonic cells were synchronized by addition of 20 mM HU (Sigma-Aldrich) during 1 h at 20°C or 25°C. Cells were washed with PBS to remove HU and resuspended in L-15 medium. At desired time points, 25 µM of EdU and 20 mM of HU were added for 10 min. Cells were fixed with 90% methanol and stored at −20°C. Cells were permeabilized with PBS containing 0.2% Triton X-100. EdU was coupled to a cleavable biotin-azide linker (Jena Biosciences) using the reagents of the Click-iT Kit (Thermo Fisher Scientific) before DNA extraction. For an estimation of the time required to replicate the entire genome, cells were isolated, synchronized using HU, released in the presence of EdU as described above, and harvested between 0 and 130 min. Cell permeabilization and Click-iT reaction to couple the EdU-labeled DNA to Alexa Fluor 647 Azide (Invitrogen) were done as described above. Cells were treated with RNase (Roche), propidium iodide (PI, Sigma-Aldrich) was added, and levels of EdU incorporation were assessed by flow cytometry (Gallios, Beckman Coulter), measuring the average intensity of the EdU in cells with incorporation normalized by the intensity in cells without incorporation. Similarly, for determining the percentage of EdU-positive cells, asynchronous cells were incubated with EdU for 30 min, and the percentage of cells with EdU incorporation was assessed by flow cytometry.

### Repli-seq

Asynchronous embryonic cells extracted from worms grown at 25°C were incubated with 25 µM EdU and fixed with 90% methanol. Fixation, Click-iT reaction, RNase treatment, and PI addition were carried out as described in the EdU-seq section. Cells were sorted according to their DNA content using a MoFlo Astrios flow sorter (Beckman Coulter) at the Flow Cytometry platform of the Medical Faculty of the University of Geneva. DNA was extracted as described in the EdU-seq section. DNA from nontreated cells was used as control.

### Sequencing of EdU-labeled DNA from EdU-seq and Repli-seq

Five to eight micrograms of DNA were sonicated with a bioruptor sonicator (Diagenode) to obtain fragments of 100–500 bp. EdU-labeled fragments were isolated as previously described (Macheret and Halazonetis 2019). The isolated DNA, as well as fragmented total DNA (control), was used for library preparation using the TruSeq ChIP Sample Prep Kit (Illumina). Libraries were sequenced using an Illumina HiSeq 2500 sequencer at the Genomics Platform of the University of Geneva.

### Data processing, domain annotation, and peak calling

Sequencing reads were mapped to the *C. elegans* reference genome WBcel215 using NovoAlign software. Subsequent data processing was done using custom Perl and R scripts (R Core Team 2017) that are available as Supplemental Code unless otherwise noted. Reads were transformed into 1000-bp bins (ChEC-seq and EdU-seq) or 10,000-bp bins (Repli-seq), and reads from biological replicates were merged. All files were normalized to the same number of reads. Experimental samples were normalized by subtracting the number of reads present in the corresponding bin in the control file. The control samples were DNA from nuclei treated with Free MNase (ChEC-seq), genomic DNA from cells treated with HU (EdU-seq), or genomic DNA from cells without treatment (Repli-seq). Data were smoothed with a sliding window averaging five consecutive bins. Genome Browser images were obtained by using the Sushi Bioconductor package in R (Phanstiel et al. 2014).

Domains of early and late replication were defined according to the signal enrichment present in early and late Repli-seq in WT samples. For peak calling, normalized and smoothed data were converted to SGA files using the “ChIP-convert” tool in the Vital-IT platform (https://ccg.epfl.ch//chipseq/), and the output SGA files were used as input for peak calling with the tool “ChIP-peak.” Peak lists from the ChEC-seq and EdU-seq experiments were merged, and duplicate peaks and peaks present in the genomic DNA sample were removed. The final list of peaks was clustered in 15 clusters using computeMatrix and plotHeatmap from deepTools (Ramírez et al. 2016). To obtain heatmaps, clusters with similar enrichment were merged together, and one cluster, containing signal only in the EdU-seq sample, was deleted, because the sites in this cluster did not show fork movement after release from the HU block and therefore likely do not represent EdU incorporation at origins. The genomic annotation (intragenic or extragenic) of early, late, and dormant origins was determined using PAVIS website (https://manticore.niehs.nih.gov/pavis2/) (Huang et al. 2013). Peak width and peak enrichment at origins were determined for each replicate separately. The peak width was measured at half height over background, and peak enrichment was defined as the difference between the signal at the peak position and the signal at random positions within a 50-kb window around the peak. For comparison of the identified origins with the ones reported in two previous studies (Pourkarimi et al. 2016; Rodríguez-Martínez et al. 2017), origins were considered as overlapping if they are located within the same 5-kb window.

### DNA combing and staining

DNA combing was performed as previously described (Michalet et al. 1997). For measuring fork speed and determining the frequency of stalled forks, embryonic cells were incubated with 20 mM HU for 1 h, 40 µM CldU for 20 min, and 400 µM IdU for 20 min at the desired temperature, with washes after each step. To determine the ability to restart DNA replication after HU treatment, embryonic cells were incubated with 40 µM CldU for 20 min, 20 mM HU for 1 h, and 400 µM IdU for 20 min, with washes after each step. Cells from both experiments were frozen at −20°C, embedded into agarose plugs, and DNA was processed using the FiberPrep DNA extraction kit (Genomic Vision). Combing and antibody incubation were done as previously described (Costantino et al. 2014). The DNA combing slides were imaged on a Leica DM5000B microscope, and the images were analyzed with Fiji software (Schindelin et al. 2012).

## Data access

All raw and processed sequencing data generated in this study have been submitted to the NCBI Gene Expression Omnibus (GEO; https://www.ncbi.nlm.nih.gov/geo/) under accession number GSE140804. The scripts used to process the data are available as Supplemental Code. The raw data used in each figure are listed in Supplemental Table S2.

## Competing interest statement

The authors declare no competing interests.

## Supplementary Material

Supplemental Material
