# Supplementary material for: Loss of histone H3.3 results in DNA replication defects and altered origin dynamics in C. elegans
Source: Genome Res. 2020 Dec;30(12):1740–51. doi: 10.1101/gr.260794.120 (PMC7706726; doi:10.1101/gr.260794.120)
Supplement: Supplemental Material [file supp_30_12_1740__index.html]

Loss of histone H3.3 results in DNA replication defects and altered origin dynamics in C. elegans — Supplemental Material 

# Loss of histone H3.3 results in DNA replication defects and altered origin dynamics in *C. elegans*

## Supplemental Material

- Supplemental\_Materials.pdf
- Supplemental\_Code.zip
