# Supplementary material for: Loss of histone H3.3 results in DNA replication defects and altered origin dynamics in C. elegans
Source: Genome Res. 2020 Dec;30(12):1740–51. doi: 10.1101/gr.260794.120 (PMC7706726; doi:10.1101/gr.260794.120)
Supplement: Supplemental Material [file supp_gr.260794.120_Supplemental_Materials.pdf]

Supplemental Materials for:

Loss of histone H3.3 results in DNA replication defects and altered origin dynamics in *C. elegans*.

Authors: Maude Strobino, Joanna M. Wenda, Laura Padayachy and Florian A. Steiner

Affiliations:

Department of Molecular Biology and Institute for Genetics and Genomics in Geneva, Section of Biology, Faculty of Sciences, University of Geneva, 1211 Geneva, Switzerland

Correspondence to: florian.steiner@unige.ch

Contents:

Supplemental Methods

Supplemental Figures S1-S7

Supplemental Tables S1-S2

Supplemental References

Supplemental Code (separate .zip file)

Supplemental Methods

Worm culture and strain generation

Worms were grown on NGM plates seeded with OP50 for maintenance, and on peptone-rich NGM plates seeded with NA22 for large-scale experiments. The sequence coding for MNase was inserted at the *cdt-1* and *tres-1* loci using CRISPR-Cas9 as described in (Arribere et al. 2014), using plasmids with 1 kb homology arms as repair templates. A list of the strains used in this study is given in Table S1. RNAi experiments were carried out by feeding, using clones from the Ahringer library (Kamath et al. 2003). For experiments measuring effects at 25°C, worms were grown at this temperature for at least 5 generations.

Phenotype analysis

For determining embryonic lethality at 25°C, L4 worms were placed on plates containing RNAi or control food overnight. Single adults were transferred to new plates for 3 h and then removed. The number of eggs, hatched larvae and adults was counted on subsequent days to determine the penetrance of embryonic lethality. Plates with 0 or 1 embryos were excluded from the final count. 30 plates (10 plates analyzed on three different days each) per experiment and condition were assessed. Total number of embryos: WT control = 445, Δ H3.3 control = 240, WT *chk-1* RNAi = 270, Δ H3.3 *chk-1* RNAi = 251.

For determining the cell cycle length during early embryonic cell divisions at 25°C, L4 worms were placed on plates containing RNAi or control food overnight. Adults were cut in egg buffer (118mM NaCl, 48 mM KCL, 2mM $CaCl_2$, 2mM $MgCl_2$ and 25mM Hepes pH7,5) to release young embryos. These embryos were mounted on 2% agarose pads and recorded every 10 s with DIC settings on a Leica DMI8 microscope, with the temperature maintained at 25°C. Cell cycle timing was measured from the appearance of the cleavage furrow of the P0 cell to nuclear envelope breakdown of the AB cell. WT control = 3, Δ H3.3 control = 5, WT RNAi = 4, Δ H3.3 RNAi = 4.

For determining the subcellular localization of CDT-1 and TRES-1, embryos cut from adult worms were stained according to standard procedures. After freeze-cracked and fixing in

cold methanol for 5 minutes, samples were incubated with anti-HA antibody (mAb 42F13, FMI Monoclonal Antibody Facility; 1:60) for HA::MNase::CDT-1 staining or anti-FLAG antibody (Sigma-Aldrich F7425.2MG; 1:200) for FLAG::MNase::TRES-1 staining, followed by incubation with secondary antibodies (Alexa Fluor 488 goat anti-mouse, Thermo Fisher Scientific A-11029, or Cy^{TM}3 donkey anti-rabbit, Jackson ImmunoResearch 711-165-152; 1:700). Samples were counterstained with DAPI and mounted in VECTASHIELD Antifade Mounting Medium. Images were acquired with Leica SP8 confocal microscope. For all the images Z-sections with 0.3 µm step were acquired, and processed with Fiji software (maximum Z-projection, contrast adjustment, Gaussian blur filter r=0.5) (Schindelin et al. 2012).

Chromatin Endogenous Cleavage (ChEC) and MNase-seq

Worm cultures were synchronized by embryo isolation with sodium hypochlorite for 4 generations. Embryos were treated with chitinase (1h, RT , 2 U/ml), washed twice in buffer A (15 mM Tris pH 7.5, 0.1 mM EGTA, 0.34 M sucrose, 0.2 mM spermine, 0.5 mM spermidine, 0.5 mM PMSF) and then resuspend in buffer A supplemented with detergents (0.25% NP-40 and 0.1% Tx-100). Nuclei were released with a glass dounce homogenizer and 15 stokes using the loose inserting pestle and 15 stokes using the tight inserting pestle. Debris were spun down at 100 g for 2 minutes and the supernatant, containing nuclei, was saved. The debris were resuspended in buffer A supplemented with detergents and the nuclei isolation step was repeated. The supernatants containing the nuclei were pooled, and the nuclei were pelleted at 1000 g for 10 minutes. Nuclei were washed once in buffer A, once in buffer B (15 mM Tris pH 7.5, 80 mM KCl, 0.1 mM EGTA, 0.2 mM spermine, 0.5 mM spermidine, 1 mM PMSF), and resuspended in buffer B. MNase was activated by the addition of $CaCl_2$ to a final concentration of 2 mM. Samples were incubated for 5 to 10 minutes (for CDT-1 ChEC), 5 to 7 minutes (for TRES-1 ChEC) or for 1 h (free MNase control) at 30°C. For the control sample, 0.3 U/ml of MNase (Biolabs, Cat. No. M0247S) was added at the same time as the $CaCl_2$. MNase was inactivated by adding an equal volume of

2x stop buffer (400 mM NaCl, 20 mM EGTA). Samples were treated overnight with Proteinase K and SDS at 55°C. Phenol-chloroform-isoamyl treatment was used to remove Proteinase K prior to RNase treatment (Roche, Cat. No. 11119915001) for 1h 30 minutes at 37°C. After chloroform extraction, DNA was precipitated overnight with ethanol and 0.25 M NaCl. DNA was resuspended overnight in TE prior to constructing the libraries using NEBNext® Ultra™ II DNA Library Prep. Fifty base pair paired-end read sequencing reactions were then performed on an Illumina HiSeq 2500 sequencer at the Genomics Platform of the University of Geneva, except for TRES-1 ChEC-seq samples from 25°C, which were sequenced with one hundred base pair single-end read sequencing reactions.

A similar protocol was used for MNase-seq, with the following modifications. Samples were incubated for different times (30s, 1min, 2min, 5min, 7min and 10min; to account for differential MNase-sensitivity of chromatin) at 30°C in presence of 2 mM $CaCl_2$ and 0.1U/ml MNase. DNA from the different times was pooled before library construction. The MNase-seq profiles around origins were determined after in silico size selection of the fragments between 120 bp and 200 bp (1_Reads_to_bins). The data were merged from 3 replicates for WT and 3 replicates for Δ H3.3.

<u>Embryonic cell extraction for EdU incorporation or DNA combing</u>

Cells were extracted from embryos as described in (Bianchi and Driscoll 2006). Briefly, embryos were isolated using sodium hypochlorite, treated with chitinase (1h, RT, 2U/ml), washed once in M9 and resuspend in 10ml of L-15 medium (supplemented with 10% FBS (heat inactivated, Gibco), 41 mM sucrose and 1% pen/strep (1:100)). Cells were dissociated with three passes through a syringe with a 22 gauge needle and two passes through a syringe with a 26 gauge needle. Debris were pelleted by spinning at 80 g for 1 min, and the supernatant, containing cells, was saved. Debris were resuspended in supplemented L-15 medium and cell extraction was repeated twice more. Supernatants were pooled together and filtered using 5 micron filters. Cells were pelleted at 500 g for 15 min and resuspended in supplemented L-15 medium.

EdU-seq

EdU-seq was carried out as described (Macheret and Halazonetis 2019) with minor modifications. Embryonic cells were synchronized by addition of 20 mM of HU (Sigma-Aldrich, Cat. No. H8627) during 1h at 20°C or 25°C. Cells were washed twice with PBS to remove HU and resuspended in L-15 medium. At desired time points, 25 μM of EdU (Invitrogen, Cat. No. A10044) and 20 mM of HU were added for 10 minutes. Cells were washed twice with PBS, fixed with 90% methanol and stored at -20°C. Cells were washed with ice-cold PBS and permeabilized with PBS containing 0.2% Triton X-100 for 30 minutes at room temperature in the dark. EdU was coupled to a cleavable biotin-azide linker (Azide-PEG(3+3)-S-S-biotin) (Jena Biosciences, Cat. No. CLK-A2112-10) using the reagents of the Click-it Kit (Invitrogen, Cat. No. C-10424). Cells were washed with PBS and treated overnight at 50°C with proteinase K in the lysis buffer. DNA was extracted by phenol/chloroform/isoamyl extraction followed by chloroform extraction. DNA was precipitated overnight at -20°C with presence of ethanol and 0.25 M NaCl and resuspended overnight in TE.

For an estimation of the time required to replicate the entire genome, cells were isolated, synchronized using HU, and released in the presence of EdU as described above. Cell aliquots were harvested between 0 and 130 min. Cell permeabilization and click-it reaction were done as described above to couple Alexa Fluor™ 647 Azide (Thermo Fisher Scientific Cat. No. A10277) to the EdU-labeled DNA. Cells were washed twice with PBS and treated with RNase (Roche, Cat. No. 11119915001) for 30 minutes at 37°C. Propidium iodide (PI, Sigma-Aldrich, Cat. No. 81845) was added, and samples incubated overnight at room temperature in the dark. Levels of EdU incorporation were assessed by flow cytometry (Gallios, Beckman Coulter), measuring the average intensity of the EdU in cells with incorporation normalized by the intensity in cells without incorporation. Similarly, for determining the percentage of EdU-positive cells, asynchronous cells were incubated with

EdU for 30 minutes, and the percentage of cells with EdU incorporation was assessed by flow cytometry.

Repli-seq

Asynchronous embryonic cells extracted from worms grown at 25°C were incubated for 5 minutes with 25 µM EdU, washed twice with PBS and fixed with 90% methanol overnight. After fixation and click-it reaction as described in the EdU-seq section, cells were washed twice with PBS and treated with RNase for 30 minutes at 37°C. PI was added, and samples were incubated overnight at room temperature in the dark. Cells were sorted according to their DNA content using a MoFlo Astrios flow sorter (Beckman Coulter) at the Flow Cytometry platform of the Medical Faculty of the University of Geneva. DNA was extracted as described in the EdU-seq section. DNA from non-treated cells was used as control.

Isolation and sequencing of EdU-labelled DNA from EdU-seq and Repli-seq

Five to eight µg of DNA were sonicated with a bioruptor sonicator (Diagenode) to obtain fragments of 100-500 bp. EdU-labelled fragments were isolated using Dynabeads MyOne Streptavidine C1 (Thermo Fisher Scientific, Cat. No. 65001) as previously described (Macheret and Halazonetis 2019). DNA was eluted by the addition of 2% B-mercaptoethanol (Sigma-Aldrich, Cat. No. M6250) and incubation for 1h at room temperature. The eluted DNA, as well as fragmented total DNA (control), was used for library preparation using the TruSeq ChIP Sample Prep Kit (Illumina, Cat. No. IP-202-1012). One hundred base pair single-end read sequencing reactions were performed on an Illumina HiSeq 2500 sequencer at the Genomics Platform of the University of Geneva.

Data processing, domain annotation and peak calling

Sequencing reads were mapped to the *C. elegans* reference genome WBcel215 using NovoAlign software (default parameters, producing SAM format files). Reads were transformed into 1’000 bp bins (ChEC-seq and EdU-seq) or 10’000 bp bins (Repli-seq)

(JSON format) with a custom perl script (1_Reads_to_bin). Reads from biological replicates were merged to obtain a single JSON file per condition (Repli-seq : WT early (2), WT mid (1), WT late (3), ∆ H3.3 early (2), ∆ H3.3 mid (2), ∆ H3.3 late (2), ChEC-seq : CDT-1 20°C (5), TRES-1 20°C (4), TRES-1 25°C (2), TRES-1 25°C ∆ H3.3 (2), EdU-seq : WT 0 min 20°C (4), all the other EdU-seq data (2)). A custom R script (2_Bed_prepa.R) was used to transform JSON files into bedgraphs (R Core Team 2017). The same R script was used to normalize all files to the same number of reads. Experimental samples were then normalized by subtracting the number of reads present in the corresponding bin in the control file. The control samples were DNA from nuclei treated with free MNase (ChEC-seq), genomic DNA from cells treated with HU (EdU-seq), or genomic DNA from cells without treatment (Repli-seq). Finally, the data were smoothed with a sliding window averaging 5 consecutive bins using a custom R script (3_Smooth_data.R). Genome Browser images were obtained by using the Sushi Bioconductor package in R (Phanstiel et al. 2014).

Domains of early and late replication were defined according to the signal enrichment present in early and late Repli-seq in WT samples. Bins with positive signal in early and negative signal in late datasets were defined as early domains, and vice versa for the ones with negative signal in early and positive signal in late as late domain. The bins with either positive signal in early and late repli-seq or negative signal in both datasets were not assigned to a domain. Domains smaller than 10 kb were merged with the previous domain (4_Domains_calling.R).

For peak calling, normalized and smoothed data were converted to SGA files using the “ChIP-convert” tool in the Vital-it platform (https://ccg.epfl.ch//chipseq/). The output SGA files were used as input for peak calling with the tool “ChIP-peak” of the Vital-it platform with these specific parameters: Cut-off = 1000, Read counts = 10, Window = 3000, Vicinity = 5000, strand = any, Repeat Masker = on, Refine peak position = off, Relative Enrichment 325 (CDT-1), 450 (TRES-1) or 350 (EdU). Peak lists from the ChEC-seq and EdU-seq experiments were merged, and duplicate peaks removed (5_remove_overlapping_peaks.R). Peaks are considered duplicate if two datasets contain a peak within the same 5kb window.

To avoid sequencing artefacts, peaks that were also enriched in the genomic DNA sample were removed. These peaks were found using computeMatrix (-bs 1000 --referencePoint center -a 5000 -b 5000 --sortRegions no) and plotHeatmap (--sortUsing sum --kmeans 20) from deepTools (Ramírez et al. 2016) . The final list of peaks was then clustered in 15 using computeMatrix (-bs 1000 --referencePoint center -a 5000 -b 5000 --sortRegions no) and plotHeatmap (-sortUsing sum --kmeans 15) from deepTools (Ramírez et al. 2016). Clusters with similar enrichment were merged together and one cluster, containing signal only in the EdU-seq sample, was deleted, as the sites in this cluster did not show fork movement after release from the HU block and therefore likely does not represent EdU incorporation at origins. Heatmaps were created using computeMatrix (-bs 1000 --referencePoint center -a 50000 -b 50000 --sortRegions no) and plotHeatmap (--sortUsing mean --missingDataColor 1) from deepTools (Ramírez et al. 2016). The genomic annotation (intragenic or extragenic) of early, late and dormant origins was determined using PAVIS website (https://manticore.niehs.nih.gov/pavis2/) (Huang et al. 2013).

Peak width and peak enrichment

Peak width and peak enrichment at origins were determined for each replicate of the datasets. The peak width was measured at half-height over background with a custom R script (6_Peaks_size.R). Peak enrichment was defined as the difference between the signal at the peak position and the signal at random positions within a 50 kb window around the peak and calculated with a custom R script (7_Peaks_enrichment.R).

Comparison with previously reported origins

The origins identified in this paper were compared to the ones identified in two previous studies (Rodríguez-Martínez et al. 2017; Pourkarimi et al. 2016). The origins in this study were considered as overlapping with previously mapped origins if they are located within the same 5 kb window. This analysis was done using a custom R script (8_compare.R).

DNA combing and staining

DNA combing was performed as described in (Michalet et al. 1997). For measuring fork speed and determining the frequency of stalled forks, embryonic cells were incubated with 20 mM HU for 1h, washed twice with ice-cold PBS, and incubated with 40 μM CldU for 20 minutes. Cells were washed twice with L-15 and then incubated in presence of 400 μM IdU for 20 minutes. To determine the ability to restart DNA replication after HU treatment, embryonic cells were incubated with 40 μM CldU for 20 minutes, washed twice with ice-cold PBS and incubated in L-15 with 20 mM HU for 1h. Cells were washed twice with L-15 and then incubated in presence of 400 μM IdU for 20 minutes. Cells from both experiments were washed with PBS and frozen at -20°C. Cells were embedded into agarose plugs, and DNA was processed using the FiberPrep® DNA extraction kit (Genomic Vision). Combing and antibody incubation were done as described in (Costantino et al. 2014). For fork speed and stalling experiments, worms and cells were kept at 20°C or 25°C, while for fork restart experiments, worms and cells were kept at 25°C. The DNA combing slides were imaged on a Leica DM5000B microscope, and the images were analyzed with Fiji software (Schindelin et al. 2012). The analysis was carried out with data from six independent experiments with a total of 36 measurements (fork speed, WT, 20°C), 17 measurements (fork speed, WT, 25°C), 15 measurements (fork speed, Δ H3.3, 20°C), 19 measurements (fork speed, Δ H3.3, 25°C), 19 measurements (fork stalling, WT, 25°C), 24 measurements (fork stalling, Δ H3.3, 25°C), 20 measurements (fork restart, WT 25°C) and 26 measurements (fork restart, Δ H3.3, 25°C).

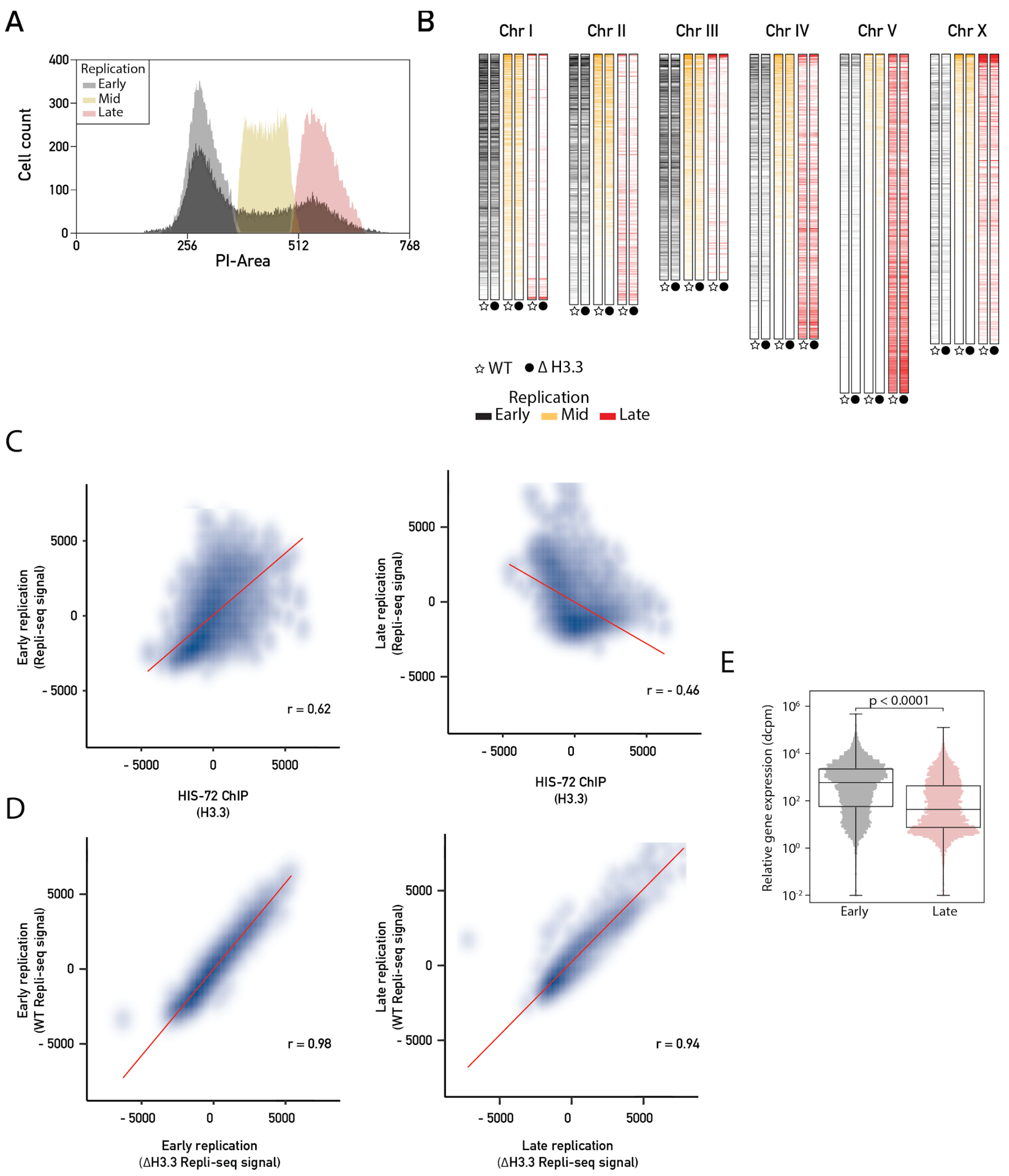

Supplemental Figure S1. Characteristics of early and late replicating domains. (**A**) Boundaries used to sort embryonic cells according to their DNA content. Plot showing the number of cells counted (cell counts) as a function of the amount of PI incorporation (PI-Area). Cells were sorted according to their DNA content into early replication (gray), mid replication (yellow) and late replication (red). (**B**) Color-coded replication timing for each chromosome. Repli-seq signal from early (black), mid (orange) and late (red) S phase for wild type (WT) and H3.3 null mutant (Δ H3.3) worms. Data is shown according to the chromosomal location. (**C**) Correlation plots between Repli-seq data for WT early (left panel)

or late (right panel) replication with HIS-72 ChIP-seq data (Delaney et al. 2019), in 10kb bins. (**D**) Correlation plots between Repli-seq data for WT and Δ H3.3 early (left panel) or late (right panel) replication, in 10kb bins. The Spearman's rank correlation coefficient (r) is shown in (C, D). (**E**) Gene expression levels for genes present in domains of early and late replication. RNA-seq data from (Kramer et al. 2015). Significance was tested using an unpaired *t*-test.

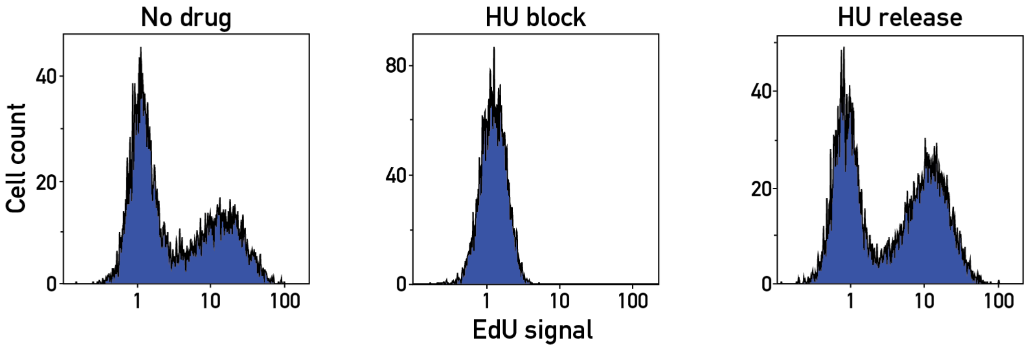

Supplemental Figure S2. Example FACS profiles of HU block experiments. Plots show distribution of EdU incorporation across cell populations in absence of HU (left panel), upon HU block (center panel) and 30 minutes after release from the HU block (right panel).

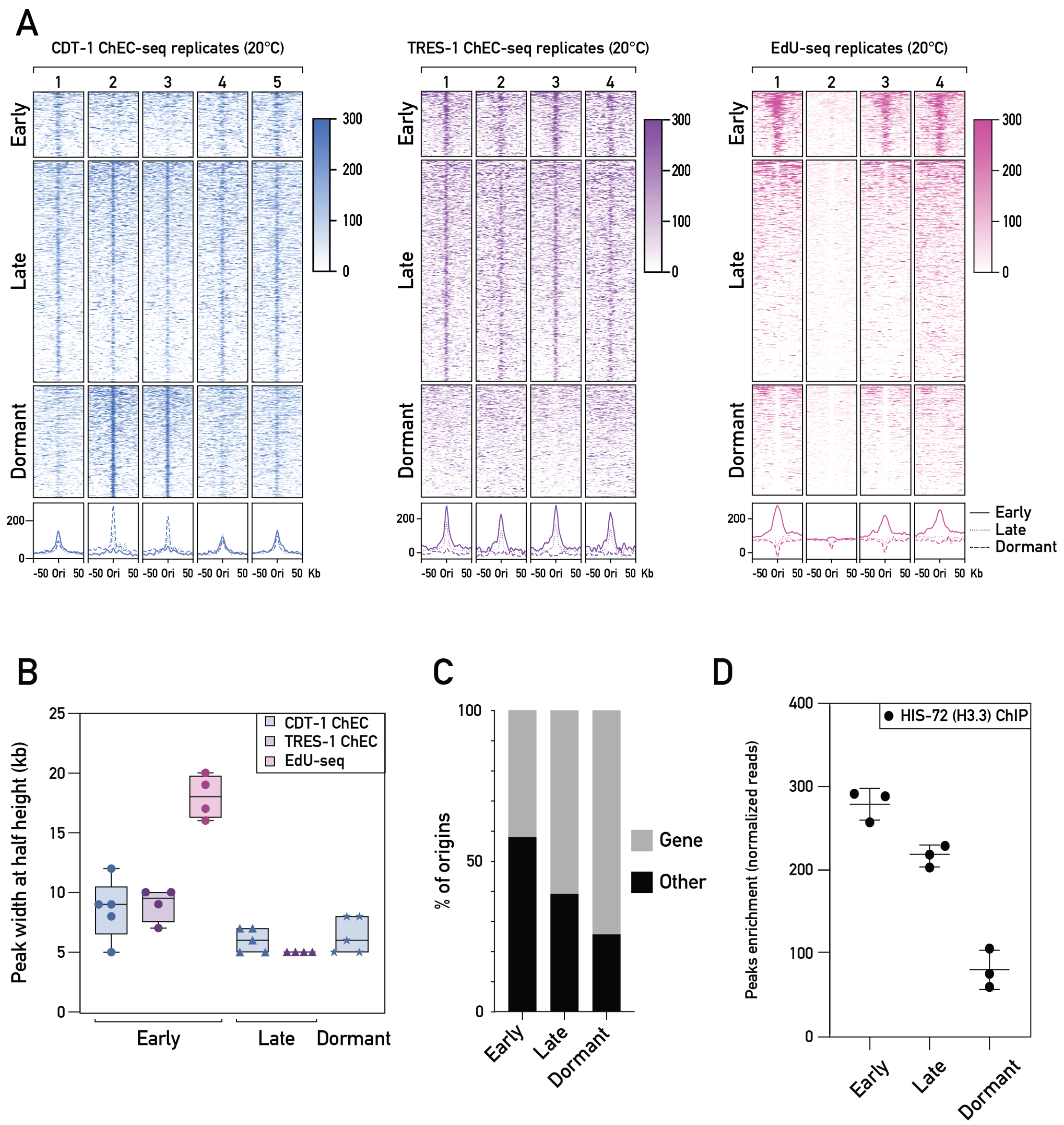

Supplemental Figure S3. Characteristics of replication origins at 20°C. (**A**) Heatmaps (top) and average plots (bottom) of replicates of CDT-1 ChEC-seq (blue), TRES-1 ChEC-seq (purple) and EdU-seq (pink) experiments at 20°C. Signal (normalized reads) is shown for a 50 kb window around each replication origin. (**B**) Peak width at half height (kb) for CDT-1 ChEC-seq (blue), TRES-1 ChEC-seq (violet) and EdU-seq (pink) signal at early, late and dormant origins. (**C**) Classification of early, late and dormant origins identified in this study based on genomic location within (genic) or outside (non-genic) gene-coding regions. (**D**) Signal enrichment quantification for H3.3 ChIP-seq data ((Delaney et al. 2019)) at early, late and dormant origins.

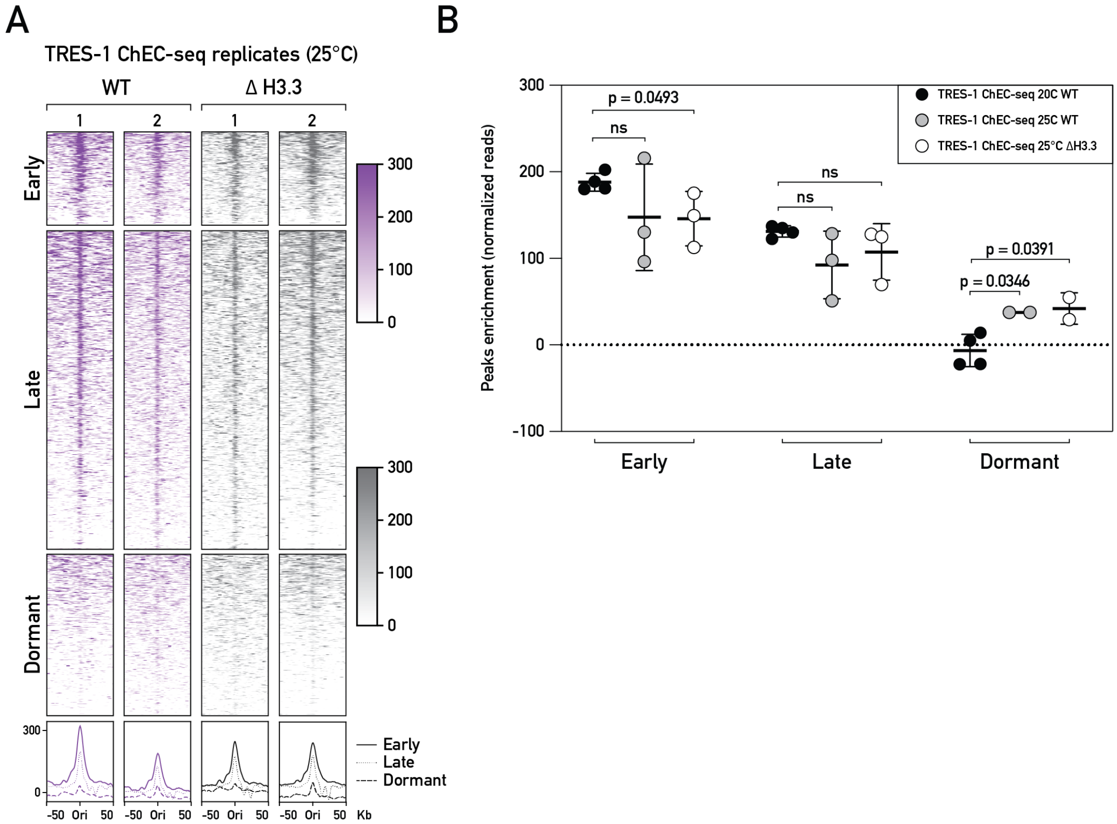

Supplemental Figure S4. TRES-1 ChEC-seq at 25°C. (**A**) Heatmaps (top) and average plots (bottom) of replicates of TRES-1 ChEC-seq experiments for wild type (WT, purple) and H3.3 null mutant (Δ H3.3, gray) worms grown at 25°C. Signal (normalized reads) is shown for a 50 kb window around each replication origin. (**B**) Signal enrichment quantification for TRES-1 ChEC-seq WT 20°C (black), TRES-1 ChEC-seq WT 25°C (gray) and TRES-1 ChEC-seq Δ H3.3 25°C (white) at early, late and dormant origins. Significance was tested using unpaired *t*-tests.

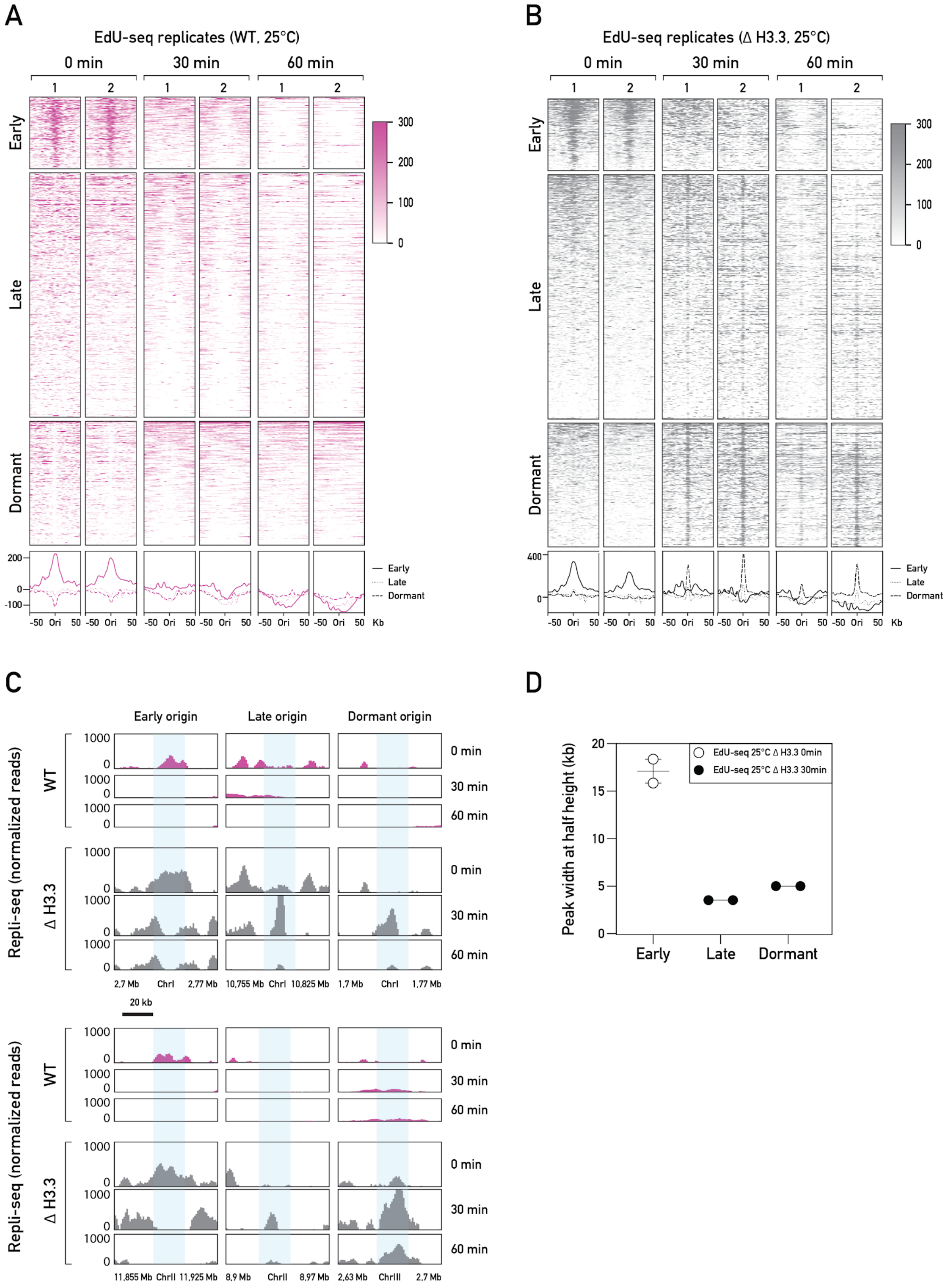

Supplemental Figure S5. EdU-seq time course at 25°C. (**A, B**) Heatmaps (top) and average plots (bottom) of replicates of EdU-seq time course experiments (0, 30 and 60 min) for wild type (A) and H3.3 null mutant (B) worms grown at 25°C. Signal (normalized reads) is shown for a 50 kb window around each replication origin. (**C**) Genome browser views of EdU-seq signal at 0, 30 and 60 minutes, showing fork progression at representative

examples of early, late and dormant origins. (**D**) Peak width at half height (kb) for EdU-seq signal at early (0 min), late (30 min) and dormant (30 min) origins in H3.3 null mutant embryos at 25°C.

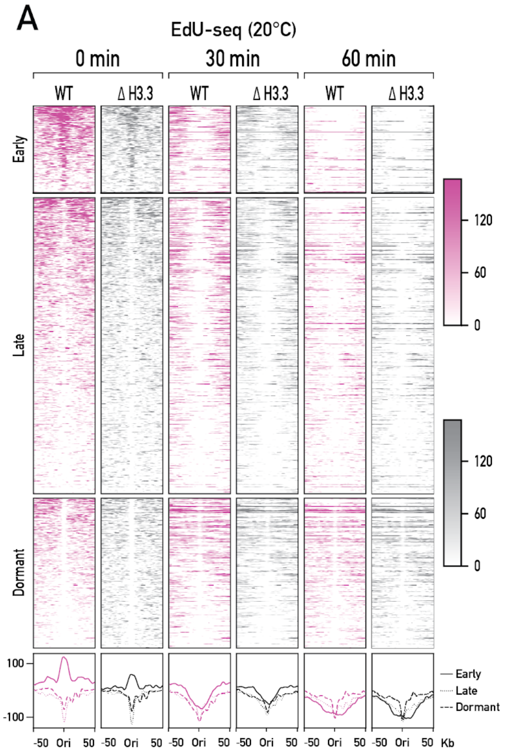

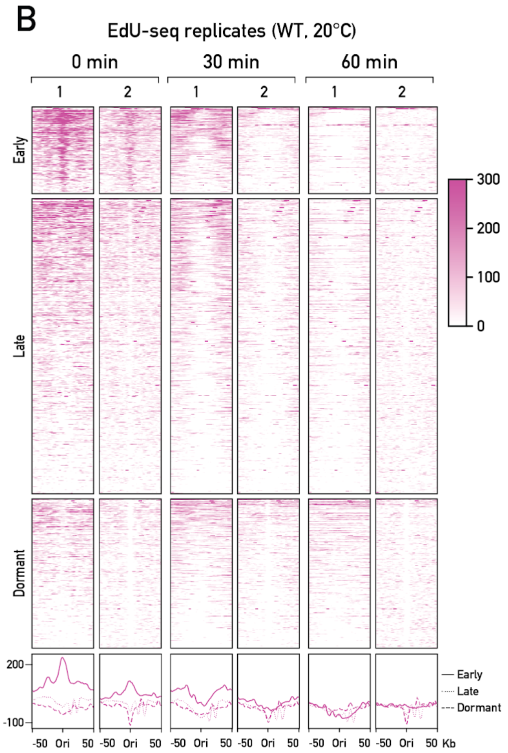

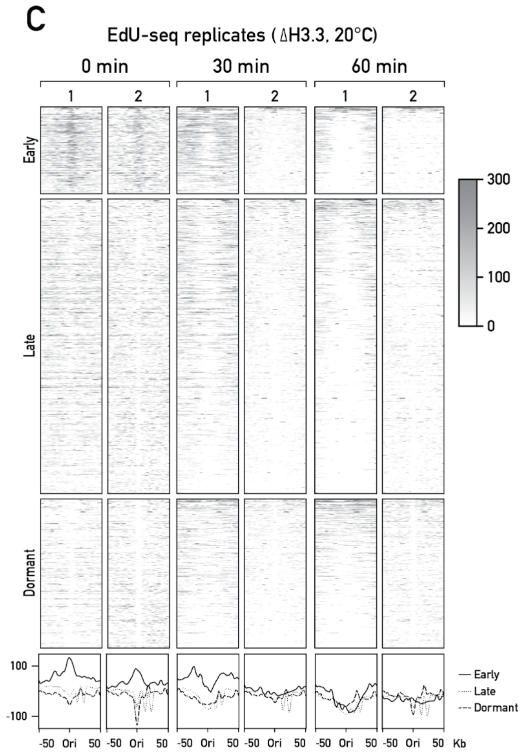

Supplemental Figure S6. EdU-seq time course at 20°C. Heatmaps (top) and average plots (bottom) of replicates of EdU-seq time course experiments (0, 30 and 60 min) for wild type (WT, pink) and H3.3 null mutant (Δ H3.3, gray) worms grown at 20°C. Signal

(normalized reads) is shown for a 50 kb window around each replication origin. (**A**) Merged data for WT and Δ H3.3. (**B**) Replicates for WT. (**C**) Replicates for Δ H3.3.

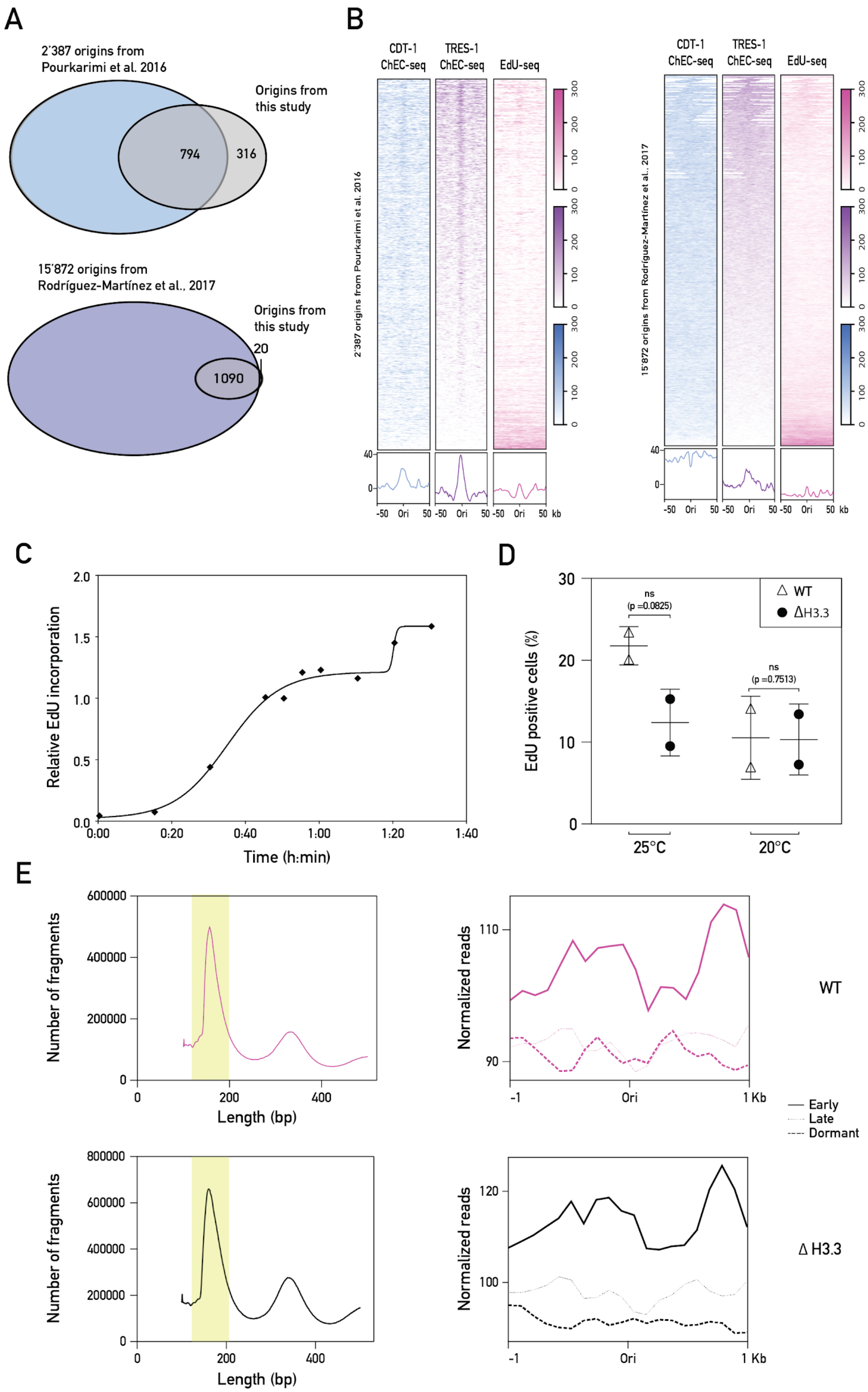
A
2'387 origins from Pourkarimi et al. 2016
Origins from this study
794
316
15'872 origins from Rodríguez-Martínez et al., 2017
Origins from this study
20
1090
B
CDT-1 ChEC-seq
TRES-1 ChEC-seq
EdU-seq
2'387 origins from Pourkarimi et al. 2016
15'872 origins from Rodríguez-Martínez et al., 2017
-50 Ori 50 kb
C
Relative EdU incorporation
Time (h:min)
D
EdU positive cells (%)
WT
ΔH3.3
ns (p =0.0825)
ns (p =0.7513)
25°C
20°C
E
Number of fragments
Length (bp)
Normalized reads
-1 Ori 1 Kb
Early
Late
Dormant
WT
Δ H3.3

Supplemental Figure S7. Validation and analysis of replication origins. (**A**) Venn diagrams representing the number of origins identified in this study that overlap with the origins found in (Pourkarimi et al. 2016) (top) and in (Rodríguez-Martínez et al. 2017) (bottom). (**B**) Heatmaps (top) and average plots (bottom) of CDT-1 ChEC-seq (blue), TRES-1 ChEC-seq (purple) and EdU-seq (pink) signal at replication origins found in (Pourkarimi et al. 2016) (left) and in (Rodríguez-Martínez et al. 2017) (right). Signal (normalized reads) is shown for a 50 kb window around each replication origin. (**C**) Time course of EdU incorporation after HU release at 20°C. Relative EdU incorporation is shown as a function of the time of incubation in presence of EdU. The curve was obtained using a bell-shaped model with GraphPad Prism version 8.0.0. (**D**) Percentage of EdU positive cells in wild type (WT, white triangles) and H3.3 null mutant (Δ H3.3, black circles) at 20°C and 25°C. Significance was tested using unpaired *t*-tests. (**E**) MNase-seq analysis of WT and Δ H3.3 embryos grown at 25°C. Left, fragment size distribution in WT and Δ H3.3 samples after MNase digest and sample pooling, indicating that MNase digest was equal for the two samples. The yellow box indicates the fragment sizes selected in silico for the analysis (120-200 bp). Right, MNase-seq profiles at replication origins in WT and Δ H3.3.

Supplemental Table S1. Overview of *C. elegans* strains used in this study

Strain	Genotype	Source	Description
N2		CGC	Wild type (WT)
FAS43	*his-69&his-70(uge44) III; his-72(tm2066) III; his-74(uge18) V; his-71(ok2289) X*	Delaney et al., 2018	H3.3 null mutant (Δ H3.3)
FAS50	*cdt-1(uge34 [HA::MNase::cdt-1]) I*	This study	CDT-1 ChEC in WT background
FAS137	*tres-1(uge102[FLAG::MNase::tres-1]) I*	This study	TRES-1 ChEC in WT background
FAS154	*tres-1(uge104[MNase::tres-1]) I; his-69&his-70(uge44) III; his-72(tm2066) III; his-74(uge18) V; his-71(ok2289) X*	This study	TRES-1 ChEC in Δ H3.3 background
FAS160	*cdt-1(uge34 [HA::MNase::cdt-1]) I; tres-1(uge102[FLAG::MNase::tres-1]) I*	This study	Immunofluorescence

Supplemental Table S2. Sequencing source data used for each figure panel.

File names refer to data deposited at the NCBI Gene Expression Omnibus (GEO; https://www.ncbi.nlm.nih.gov/geo/) under accession number GSE140804.

**Figure**	**Method**	**Data**
Figure 1 (B-C)	Repli-seq	Repliseq_WT_Early_final.bed<br>Repliseq_WT_mid_final.bed<br>Repliseq_WT_Late_final.bed<br>Repliseq_H3_Early_final.bed<br>Repliseq_H3_mid_final.bed<br>Repliseq_H3_Late_final.bed
Figure 2D	ChEC-seq<br>EdU-seq	CDT1_20C_final.bed<br>TRES1_20C_final.bed<br>EdU_20C_final.bed<br>Repliseq_Domains.bed
Figure 3A	ChEC-seq	CDT1_20C_final.bed<br>TRES1_20C_final.bed<br>EdU_20C_final.bed<br>Origins_Clustered.bed
Figure 3B	ChIP-seq (published data)	(Delaney et al. 2019)<br>Origins_Clustered.bed
Figure 4A	ChEC-seq	TRES1_25C_WT_final.bed<br>H3_TRES1_25C_final.bed<br>Origins_Clustered.bed
Figure 4 (B-C)	EdU-seq fork progression	EdU_WT_Fork_25C_0min_final.bed<br>EdU_WT_Fork_25C_30min_final.bed<br>EdU_WT_Fork_25C_60min_final.bed<br>H3_EdU_Fork_25C_0min_final.bed<br>H3_EdU_Fork_25C_30min_final.bed<br>H3_EdU_Fork_25C_60min_final.bed<br>Origins_Clustered.bed
Figure S1 B	Repli-seq	Repliseq_WT_Early_final.bed<br>Repliseq_WT_mid_final.bed<br>Repliseq_WT_Late_final.bed<br>Repliseq_H3_Early_final.bed<br>Repliseq_H3_mid_final.bed<br>Repliseq_H3_Late_final.bed
Figure S1 C	Repli-seq	Repliseq_WT_Early_final.bed<br>Repliseq_WT_Late_final.bed<br>Delaney et al., 2019
Figure S1 D	Repli-seq	Repliseq_WT_Early_final.bed<br>Repliseq_WT_Late_final.bed<br>Repliseq_H3_Early_final.bed<br>Repliseq_H3_Late_final.bed
Figure S3 A-B	ChEC-seq	Bed files for individual replicates

	EdU-seq	Origins_Clustered.bed
Figure S3 D	-	(Delaney et al. 2019)<br>Origins_Clustered.bed
Figure S4	ChEC-seq	Bed files for individual replicates of TRES-1 ChEC-seq at 25°C and 20°C<br>Origins_Clustered.bed
Figure S5	EdU-seq fork progression	Bed files for individual replicates of EdU-seq fork progression at 25°C<br>Origins_Clustered.bed
Figure S6	EdU-seq fork progression	EdU_WT_Fork_20C_0min_final.bed<br>EdU_WT_Fork_20C_30min_final.bed<br>EdU_WT_Fork_20C_60min_final.bed<br>H3_EdU_Fork_20C_0min_final.bed<br>H3_EdU_Fork_20C_30min_final.bed<br>H3_EdU_Fork_20C_60min_final.bed<br>Bed files for individual replicates of EdU-seq fork progression at 20°C<br>Origins_Clustered.bed
Figure S7 A-B	-	Origins_Clustered.bed<br>(Rodríguez-Martínez et al. 2017)<br>(Pourkarimi et al. 2016)
Figure S7 E	MNase-seq	N2_MNase.bed<br>H3_MNase.bed

Supplemental References

Arribere JA, Bell RT, Fu BXH, Artiles KL, Hartman PS, Fire AZ. 2014. Efficient marker-free recovery of custom genetic modifications with CRISPR/Cas9 in Caenorhabditis elegans. *Genetics* **198**: 837–846.

Bianchi L, Driscoll M. 2006. Culture of embryonic C. elegans cells for electrophysiological and pharmacological analyses. *WormBook* 1–15.

Costantino L, Sotiriou SK, Rantala JK, Magin S, Mladenov E, Helleday T, Haber JE, Iliakis G, Kallioniemi OP, Halazonetis TD. 2014. Break-induced replication repair of damaged forks induces genomic duplications in human cells. *Science* **343**: 88–91.

Delaney K, Strobino M, Wenda JM, Pankowski A, Steiner FA. 2019. H3.3K27M-induced chromatin changes drive ectopic replication through misregulation of the JNK pathway in C. elegans. *Nat Commun* **10**: 1–15.

Huang W, Loganantharaj R, Schroeder B, Fargo D, Li L. 2013. PAVIS: a tool for Peak Annotation and Visualization. *Bioinformatics* **29**: 3097–3099.

Kamath RS, Fraser AG, Dong Y, Poulin G, Durbin R, Gotta M, Kanapin A, Le Bot N, Moreno S, Sohrmann M, et al. 2003. Systematic functional analysis of the Caenorhabditis elegans genome using RNAi. *Nature* **421**: 231–237.

Kramer M, Kranz A-L, Su A, Winterkorn LH, Albritton SE, Ercan S. 2015. Developmental Dynamics of X-Chromosome Dosage Compensation by the DCC and H4K20me1 in C. elegans. *PLoS Genet* **11**: e1005698.

Macheret M, Halazonetis TD. 2019. Monitoring early S-phase origin firing and replication fork movement by sequencing nascent DNA from synchronized cells. *Nat Protoc* **14**: 51–67.

Michalet X, Ekong R, Fougerousse F, Rousseaux S, Schurra C, Hornigold N, van Slegtenhorst M, Wolfe J, Povey S, Beckmann JS, et al. 1997. Dynamic molecular combing: stretching the whole human genome for high-resolution studies. *Science* **277**: 1518–1523.

Phanstiel DH, Boyle AP, Araya CL, Snyder MP. 2014. Sushi.R: flexible, quantitative and integrative genomic visualizations for publication-quality multi-panel figures. *Bioinformatics* **30**: 2808–2810.

Pourkarimi E, Bellush JM, Whitehouse I. 2016. Spatiotemporal coupling and decoupling of gene transcription with DNA replication origins during embryogenesis in. *Elife* **5**. http://dx.doi.org/10.7554/eLife.21728.

R Core Team. 2017. R: A language and environment for statistical computing. R Foundation for Statistical Computing, Vienna, Austria. URL https://www.R-project.org/.

Ramírez F, Ryan DP, Grüning B, Bhardwaj V, Kilpert F, Richter AS, Heyne S, Dündar F, Manke T. 2016. deepTools2: a next generation web server for deep-sequencing data analysis. *Nucleic Acids Res* **44**: W160–5.

Rodríguez-Martínez M, Pinzón N, Ghommidh C, Beyne E, Seitz H, Cayrou C, Méchali M. 2017. The gastrula transition reorganizes replication-origin selection in Caenorhabditis elegans. *Nat Struct Mol Biol* **24**: 290–299.

Schindelin J, Arganda-Carreras I, Frise E, Kaynig V, Longair M, Pietzsch T, Preibisch S, Rueden C, Saalfeld S, Schmid B, et al. 2012. Fiji: an open-source platform for biological-image analysis. *Nat Methods* **9**: 676–682.
